# Induced Pluripotent Stem Cells as a Tool for Modeling Hematologic Disorders and as a Potential Source for Cell-Based Therapies

**DOI:** 10.3390/cells10113250

**Published:** 2021-11-19

**Authors:** Ponthip Pratumkaew, Surapol Issaragrisil, Sudjit Luanpitpong

**Affiliations:** 1 Siriraj Center of Excellence for Stem Cell Research, Faculty of Medicine Siriraj Hospital, Mahidol University, Bangkok 10700, Thailand; ponthip.pot@gmail.com (P.P.); surapol.iss@mahidol.ac.th (S.I.); 2 Division of Hematology, Department of Medicine, Faculty of Medicine Siriraj Hospital, Mahidol University, Bangkok 10700, Thailand; 3 Bangkok Hematology Center, Wattanosoth Hospital, BDMS Center of Excellence for Cancer, Bangkok 10310, Thailand

**Keywords:** induced pluripotent stem cells, disease modeling, blood disorders, genetic disorders, cell-based therapy

## Abstract

The breakthrough in human induced pluripotent stem cells (hiPSCs) has revolutionized the field of biomedical and pharmaceutical research and opened up vast opportunities for drug discovery and regenerative medicine, especially when combined with gene-editing technology. Numerous healthy and patient-derived hiPSCs for human disease modeling have been established, enabling mechanistic studies of pathogenesis, platforms for preclinical drug screening, and the development of novel therapeutic targets/approaches. Additionally, hiPSCs hold great promise for cell-based therapy, serving as an attractive cell source for generating stem/progenitor cells or functional differentiated cells for degenerative diseases, due to their unlimited proliferative capacity, pluripotency, and ethical acceptability. In this review, we provide an overview of hiPSCs and their utility in the study of hematologic disorders through hematopoietic differentiation. We highlight recent hereditary and acquired genetic hematologic disease modeling with patient-specific iPSCs, and discuss their applications as instrumental drug screening tools. The clinical applications of hiPSCs in cell-based therapy, including the next-generation cancer immunotherapy, are provided. Lastly, we discuss the current challenges that need to be addressed to fulfill the validity of hiPSC-based disease modeling and future perspectives of hiPSCs in the field of hematology.

## 1. Introduction

Over a decade ago, human cellular reprogramming from adult somatic cells to pluripotent state, so-called human induced pluripotent stem cells (hiPSCs) was successfully discovered [1,2]. Due to their unique properties of unlimited self-renewal and the ability to differentiate into all cell types of three primordial germ layers, termed ‘pluripotency’, hiPSCs offer numerous opportunities for a wide range of research and clinical applications. In particular, the groundbreaking discovery of hiPSCs has revolutionized the field of biomedical research and the study of human physiology and diseases, which ideally require that the studied cells and tissues are obtained directly from patients. In some cases, primary human cells or tissues are not easily accessible, e.g., those from the brain and heart; hence, research with certain human materials is limited. One of the merits of using hiPSCs is the potential to overcome this limited donor accessibility that, together with differentiation techniques, can establish an appropriate model system for developmental and disease studies at various pathological events from an early stage of disease progression, otherwise unavailable in primary cells. Importantly, hiPSCs that are derived from patients with genetic diseases, termed ‘patient-specific iPSCs’ or ‘disease-specific iPSCs’, contain the same genetic information as the patients, making them reliable sources for modeling human diseases. To date, some information on the pathogenesis of human diseases has been uncovered using hiPSC-based models, for example, neurodegenerative diseases, cardiac channelopathy, and muscular dystrophy [3,4,5]. Furthermore, hiPSCs also hold great promise for therapeutic purposes as a platform for drug discovery and cell-based therapy. For hematologic disorders, which have been at the forefront of biomedical research mainly due to the availability of hematopoietic stem/progenitor cells (HSPCs) in the bone marrow, the emergence of hiPSCs mainly benefits the study of genetic diseases as HSPCs generally hinder genetic modifications. In this review, we summarize the uses of hiPSCs as a research tool for hematologic disorders and highlight recent hereditary and acquired hematologic disease modeling with patient-specific iPSCs. We also provide current applications of hiPSCs as instrumental drug screening and potential therapeutic candidates for hematologic disorders, with or without further genetic corrections or modifications. Lastly, we highlight and analyze current challenges, considerations, and future research and clinical directions. 

## 2. Discovery and Development of hiPSCs

Generally, hiPSCs were generated from the reprogramming of somatic cells by ectopic expression of a set of transcription factors that converted them into unique cells with pluripotency. The reprogramming process silences the somatic gene expression and activates pluripotency regulatory network. In 2006, iPSCs were first successfully established from mouse fibroblasts and a year later from human fibroblasts [1,2,6]. Initially, Takahashi and Yamanaka selected 24 embryonic transcription factors and performed repeated retroviral transduction experiments to narrow down the pooled factors that were required to induce pluripotency in adult somatic cells—A reprogramming cocktail OCT4, SOX2, KLF4, and c-MYC (OSKM) was ultimately defined [6]. The characteristics of hiPSCs are similar to those of human embryonic stem cells (hESCs), the prototypical PSCs, which were derived from pre-implantation embryo, but largely depend on the selection of clones [7]. Both cell types are capable of unlimited proliferation and sustained self-renewal while maintaining pluripotency [1,8]. hiPSCs can be derived from various somatic cell sources compared with hESCs that are from a restricted source of the inner cell mass of a blastocyst and may involve the ethical objection of the use of human embryos in their derivation. Therefore, hiPSCs are relatively more accessible and largely used in stem cell research. The establishment of hiPSCs mainly relies on different components, including the reprogramming factors, delivery method, starting cell type, culture condition, and type of application, which should be considered prior to its generation. For basic research, high reprogramming efficiency is preferred, while the safety of the method is less of a concern. In contrast, safety and quality aspects of clinical-grade products are the first priority for clinical applications of hiPSCs. Besides undergoing transcriptional changes, the reprogramming process ideally requires the resetting of the epigenetic landscape of starting cells [9], resembling that of early development. However, studies have shown that many hiPSCs retain epigenetic memory, particularly at the low passage [10,11,12,13], which is considered to be an unresolved issue with hiPSCs compared with hESCs, although there is evidence showing that the memory could be reset by serial reprogramming or treatment with chromatin modifying compounds [13]. Remarkably, epigenetic memory may be advantageous for cell replacement therapy, as it could predispose hiPSCs to differentiate more readily into their parental cells, thus improving the differentiation efficiency.

Considering that reprogramming factors play a critical role in the regulation of cellular identity, many efforts have been made to generate hiPSCs using different cocktails of defined factors. Yu and colleagues reported the use of an alternative combination of OCT4, SOX2, NANOG and LIN28 (OSNL), identified through an independent screen of candidate genes [2]. The union of OSNL and OSKM, yielding six reprogramming factors, were shown to improve OSKM-mediated reprogramming from the synergistic activation of LIN41 and canonical WNT/β-catenin signaling by NANOG and LIN28 [14]. In some other studies, additional genes or enhancers, either small molecules or chemicals, were applied to increase reprogramming efficiency. For example, undifferentiated embryonic cell transcription factor 1 (UTF1), T-box3 transcription factor TBX3, siRNA or shRNA against p53, histone deacetylase inhibitor valproic acid (VPA), and vitamin C have been reported to enhance hiPSC generation [15,16,17,18,19,20,21]. On the other hand, several studies have attempted to simplify the reprogramming process by minimizing reprogramming factors, e.g., from OSKM to only OCT4 and SOX2 [20] or OCT4 alone [22], albeit with lower efficiency.

The efficiency of hiPSC generation is influenced greatly by the delivery method of reprogramming factors. Generally, the reprogramming efficiency of human somatic cells to hiPSCs even with viral-based method is limited, i.e., up to ~2% [23]—only a small number of transduced cells can complete molecular events and become fully reprogrammed iPSCs [24]. Concerning their safety profile, delivery systems are generally classified into two main types based on their effects on genomic integration of transgenes. Integration systems, such as retroviral and lentiviral-based vectors, were used in early studies to establish mouse and human iPSCs from various cell types [1,2,6,25]. These viral inductions provide stable transgene expression and more efficient iPSC derivation, when compared to plasmid transfection and, as predicted, non-integration systems [24]. Notably, the lentiviral transduction has advantages over the retroviral transduction since it can infect both dividing and nondividing cells, giving higher reprogramming efficiency. However, the risks of insertional mutagenesis and transgene reactivation remain their drawbacks. Shortly thereafter, several integration-free systems were developed to facilitate future clinical applications. These integration-free methods can be subdivided into: (i) viral-based vectors, e.g., adenovirus and Sendai virus (SeV); (ii) DNA-based vectors, e.g., episomal plasmids (Epi) and minicircle DNA; (iii) self-excision vectors, e.g., piggyBac transposon; (iv) modified mRNA; and (v) recombinant proteins. A previous study performed systemic evaluation on the most commonly used integration-free methods: SeV, Epi, and mRNA, and reported different efficacies and advantages among them. SeV reprogramming offers relatively high efficiency (0.01–1%) and is a reliable method [26]; it requires a smaller workload and was validated in multiple cell types, including fibroblasts and blood cells, e.g., peripheral blood mononuclear cells (PBMCs), CD34^+^ cells, and T cells for both research- and clinical-grade iPSC generation [27,28,29,30,31,32]. For Epi reprogramming, it is also highly reliable and feasible to generate clinical hiPSCs under current good manufacturing practice (cGMP), making it suitable for transition to clinical use [26,32]. mRNA reprogramming offers the highest efficiency (up to 4%) and complete absence of integration [33,34]; however, this technique is laborious mainly due to the requirement of multiple transfections. It is worth noting that the cell source used for mRNA reprogramming was mainly fibroblasts; hence, the information in blood cells is very limited [26,35]. Additionally, numerous reports have described the pivotal role of specific microRNAs (miRNAs) in reprogramming. Several groups of miRNAs have been identified as being uniquely expressed in ESCs when compared to differentiated cells. Among them, the miR-302/367 cluster has gained much attention as its expression was highly expressed in the early embryo and then rapidly decreased upon differentiation. miR-302/367 was shown to greatly improve the somatic cell reprogramming mediated by OSKM and OSK [36,37]. Anokye-Danso and colleagues reported that miR-302/367 was able to reprogram human fibroblasts even without other exogenous transcription factors [38]. However, there is still an unmet demand for optimal protocols for mRNA or miRNA reprogramming in blood cells. Although hiPSCs can be theoretically derived from any specific cell types, the starting cells with reported limited reprogramming efficiency include terminally differentiated blood cells, i.e., T cells, B cells, macrophages, and granulocytes [39]. 

The reprogramming process also involves culture conditions. Initially, a feeder system, such as inactivated mouse embryonic fibroblasts (MEFs), was used upon reprogramming to generate and maintain hESCs, and was later applied to hiPSCs. Subsequently, a feeder-free condition was established to make hiPSCs more applicable for clinical use. There have been different studies on the combinations of extracellular matrices and culture media to create a xeno-free condition for reliable propagation of hiPSCs, for example, the culture of hiPSCs on a vitronectin- or recombinant laminin-511 E8 fragment (rLN511E8)-coated surface instead of Matrigel coating in defined xeno-free media, e.g., E8 or mTeSR1, to create a xeno-free condition for clinical compliance [40,41,42]. It should be noted that optimization of these culture conditions is required for the cell type-to-cell-type basis. 

In an hiPSC-based disease model, particularly for genetic disorders, the type of starting cells is a major consideration. In hereditary genetic diseases where all cells carry the disease-causing mutations, the choice of cell types is more diverse compared with acquired genetic disorders. The cell type of the latter is restricted because acquired somatic mutations only affect certain non-germline cells, for example, only hematopoietic cells are affected in acquired hematologic diseases. Thus, the choice of starting cells is greatly important, as it represents genetic information or disease-associated mutations of the donor cells. Furthermore, identifying the disease phenotypes is also the key advantage of iPSC-based disease modeling. This strategy requires an appropriate cell differentiation protocol to produce disease-relevant cells and the development of well-control cell line used to compare the molecular and cellular phenotypes. As mentioned above, each hiPSC line may retain epigenetic memory from incomplete reprogramming. In this case, it causes line-to-line variation that may impact the data interpretation and differentiation process. However, this hurdle can be overcome by using genome editing technology to create isogenic pairs, by either correction of the disease-associated mutations or introduction of mutation in normal control iPSCs (see the following sections).

## 3. Hematopoiesis and Hematopoietic Differentiation from hiPSCs

**Figure 1:**
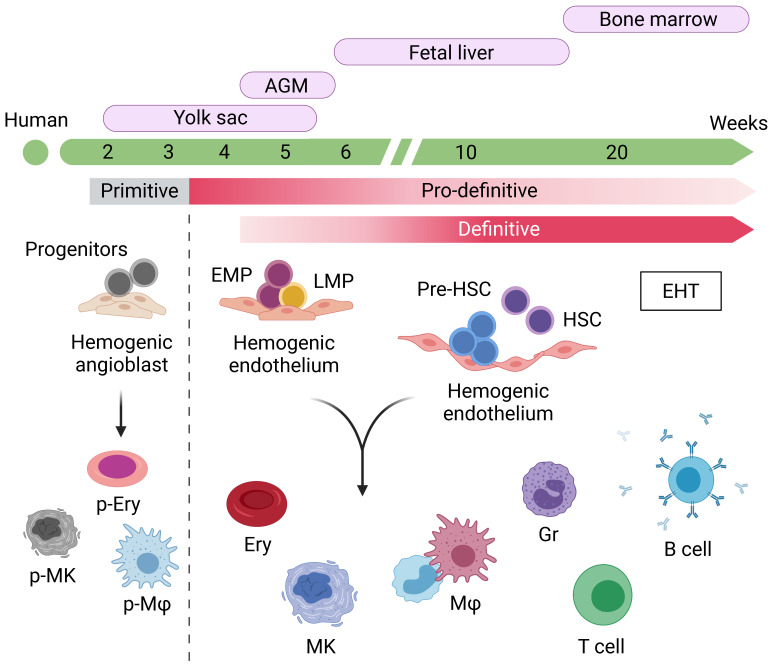
Schematic representation of human hematopoietic development. Embryonic hematopoiesis is established in spatiotemporally overlapping waves, categorized into three main waves termed primitive, pro-definitive, and definitive hematopoiesis. AGM, aorta-gonad-mesonephros; EHT, endothelial-to-hematopoietic transition; EMP, erythro-myeloid progenitor; LMP, lympho-myeloid progenitor; HSC, hematopoietic stem cell; Ery, erythrocyte; MK, megakaryocyte; Mφ, macrophage; Gr, granulocyte.

The differentiation potential of hiPSCs into any clinically relevant cell types offers great promise and opportunities for cell-based therapy and regenerative medicine, and also offers a unique chance to model human disease pathology and drug screening. Several attempts have been made to develop blood cells from hiPSCs based on the findings on embryonic hematopoiesis studied in ESCs, which were established two decades ago, or animal models. Generally, developmental hematopoiesis occurs as spatiotemporally overlapping waves and can be categorized into three main waves based on the characteristics: (i) primitive hematopoiesis, (ii) pro-definitive hematopoiesis, and (iii) definitive hematopoiesis. The first wave appears in the yolk sac and gives rise to primitive blood cells, including nucleated erythrocytes, megakaryocytes, and macrophages, but lacks lymphoid potential [43,44,45]—it provides the growth needs and innate defense mechanisms for the embryo (Figure 1).

The second wave is where adult-like blood cells emerge. In this wave, the erythro-myeloid progenitors (EMPs) originate from the hemogenic endothelium (HE) in the yolk sac and develop into erythrocytes, megakaryocytes, and myeloid lineages and subsequently migrate to the fetal liver. The second wave also produces lympho-myeloid progenitors (LMPs), which are undetectable in the primitive wave [46,47,48,49]. However, the cells in this transient wave have no long-term repopulating potential and cannot reconstitute hematopoiesis. Following this program, the first definitive hematopoietic stem cells (HSCs) emerge in the aorta-gonad-mesonephros (AGM) region, a potent hematopoietic site within the mammalian embryo body, through the endothelial-to-hematopoietic transition (EHT) process and localize in the fetal liver, where they propagate before homing to the bone marrow [50,51,52]. These definitive HSCs have multilineage differentiation and long-term engraftment potential as well as the capability of life-long hematopoiesis in adult life [52,53]. Hence, the establishment of definitive HSCs in vitro has gained much attention in the field of hematology and is a promising approach for future regenerative medicine.

**Figure 2:**
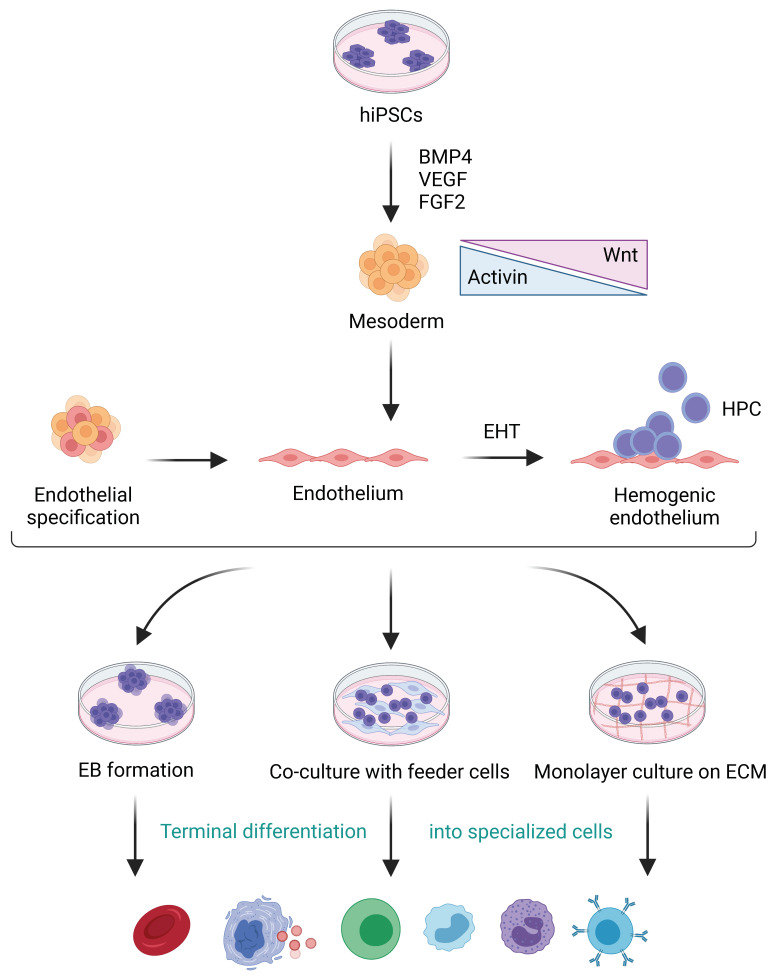
In vitro hematopoietic differentiation from hiPSCs via mesoderm induction. hiPSC, human induced pluripotent stem cell; BMP4, bone morphogenetic protein 4; VEGF, vascular endothelial growth factor; FGF2, fibroblast growth factor 2; EHT, endothelial-to-hematopoietic transition; HPC, hematopoietic progenitor cell; EB, embryoid body; ECM, extracellular matrix.

The process of hematopoiesis is associated with a dynamic regulation of various factors such as transcription factors, signaling pathways, microenvironment called the niche, and interaction between the cells. Understanding these factors enables the efficient production of blood lineages and can be, ultimately, translated to clinical used. It has been shown that the production of blood lineage from hPSCs can recapitulate early blood development during ontogeny. Several studies have demonstrated different strategies for in vitro hematopoietic differentiation from hiPSCs. The common process of these procedures begins with the differentiation of hiPSCs to exit the pluripotent state and move toward intermediate cell states such as the mesoderm and hemogenic endothelium, followed by a terminal differentiation into specialized blood cells of interest (Figure 2).

Previous studies have demonstrated that bone morphogenetic protein 4 (BMP4), fibroblast growth factor 2 (FGF2), WNT/β-catenin, and Activin/Nodal play critical roles for mesoderm formation [54,55,56,57]. Thus, specific hematopoietic growth factors and cytokines, i.e., BMP4, Activin A, CHIR99021 (GSK-3 inhibitor), SB-431542 (TGFβ inhibitor), vascular endothelial growth factor (VEGF), FGF2, stem cell factor (SCF), thrombopoietin (TPO), interleukin (IL)-3, and IL-6, were added throughout the differentiation process to induce HSC specification. In addition, lineage specification markers have been described to specify different cell populations occurring upon hematopoietic differentiation. For example, KDR, apelin receptor (APLNR), and platelet-derived growth factor receptor alpha (PDGFRα) have been used for the emergence detection of the mesoderm [58]. Some studies have used the marker KDR^+^ CD235a^−^ to identify mesodermal cells that give rise to a definitive hematopoietic program [59]. The hematopoietic induction system could be classified into three approaches: (i) the formation of embryoid bodies (EBs), of which the cells form a 3D structure that allows cell–cell interaction, mimicking the spatial organization of the embryo; (ii) co-culture with stromal feeder cells such as OP9 cells, which provides an intimate cell contact with secreting factors that promote cell proliferation and differentiation; and (iii) a 2D monolayer culture on an extracellular matrix (ECM)-coated dish that together with hematopoietic cytokine cocktails could generate a xeno-free culture [58,60,61]. Although early studies demonstrated mostly primitive HSC production from hiPSCs, recent studies have revealed improved methods by which to develop hiPSC-derived definitive HSCs, which are characterized by the presence of adult globin expression in erythrocytes, multilineage differentiation including T lymphocytes, and long-term engraftment ability in immunocompromised mice models [60,62]. Transcription factors, e.g., HOXA9, RUNX1a, and MLL-AF4, were shown to enhance hematopoietic commitment, multilineage differentiation, and engraftable HSPCs [63,64,65,66]. Although MLL-AF4 was sufficient to re-specify iPSCs into long-term engrafting CD34^+^CD38^−^CD90^+^CD49f^+^ HSPCs in immunodeficient mice, reconstituting long-term B, T, erythroid, and myeloid cells, MLL-AF4-induced HSPCs were prone to leukemic transformation during the long-term posttransplant period [66]. The efficient production of definitive HSCs would offer more clinical potential for transplantation and the production of functional bloods for cell replacement therapy and immunotherapy, yet optimal differentiation protocols remain to be elucidated. Notably, in vivo HSC differentiation from hiPSCs via teratoma formation has also been proposed to generate functional HSCs and long-term reconstitution, as this system was suggested to provide the microenvironment that resembled HSC niche [67,68,69]. However, it should be noted that specific cell types obtained from teratomas require thorough isolation prior to use, to avoid the contamination of other cells, and that this system was performed in animals; hence, this approach might be impractical for clinical use.

hiPSC-derived HSCs/HSPCs have the potential to develop further into various blood cell types: red blood cells (RBCs), megakaryocytes (MKs), myeloid cells, and lymphoid cells via terminal differentiation. For erythropoiesis, studies of hiPSC-derived RBCs have been ongoing for many years. It has been noted that RBCs differentiated from iPSCs are likely to resemble yolk sac primitive/definitive and fetal liver EMP-erythropoiesis because they have a low enucleation potential and embryonic/fetal globin expression. Several attempts have been made to overcome these hurdles by adding small molecules, e.g., inhibitor VIII (specific GSK-3β inhibitor) and 3-isobutyl-1-methylxanthine (IBMX) (cAMP inhibitor) [70], in the culture system, pre-selection definitive erythroid population [71], and manipulation of specific genes involved in erythropoiesis, e.g., *c-MYB*, *BCL11A*, *KLF1*, and *SH2B3* [72,73,74,75]. However, the enucleation rate remains slightly improved and the hemoglobin content in hiPSC-derived RBCs is partially expressed adult globin [76,77,78]. In terms of cell expansion, hiPSC-derived RBCs showed a lower expansion rate in comparison to RBCs derived from cord blood/mobilized peripheral blood [79]. Therefore, the development of RBCs from hiPSCs requires more extensive studies to improve the quality and quantity of produced RBCs. For megakaryopoiesis, hiPSCs have been differentiated into megakaryocyte lineages with various improved methods, i.e., supplementation of platelet stimulation by TPO or agonist to c-MPL (TPO receptor) in the culture, downregulation of c-MYC by specific compound iBET151, and exogenous expression of GATA1, FLI1, and TAL1 in hiPSCs, to increase MK expansion and maturation [80,81,82]. The major challenge of hiPSC-derived MKs is the generation of sufficient functional pro-platelets that might be used in clinical applications. It has been shown that culture conditions with shear stress enhanced platelet production from hiPSC-derived MKs and could be further scaled up in a bioreactor [83,84]. This progress paves the way to produce high-yield and functional platelets in vitro, and to utilize hiPSC-derived platelets in blood transfusion. The first-in-human clinical trial for hiPSC-derived platelets was demonstrated in an aplastic anemia patient who suffered from platelet transfusion refractoriness. The study used autologous iPSC-derived platelets to treat the patient and there was no evidence of an alloimmune response [85]. For the myeloid lineage, hiPSC-derived macrophages or monocytes have been more widely studied in this field due to their similarity to embryonic development that produces tissue-resident macrophages [86]. Furthermore, hiPSC-derived macrophages have been described as having similar transcriptomic profile to tissue-resident macrophages, which are normally difficult to access [87]. Previous studies demonstrated the capability of the differentiation of hiPSCs toward monocytes and functional macrophages, and this could extend to a large-scale production [88]. These systems can be applied for disease modeling, cellular therapy, and drug screening. For lymphoid cells, scholars have reported the generation of hiPSC-derived T cells through co-culturing on delta-like (DL)-1 or DL-4 expressing OP9 cells and in vivo teratoma formation [89,90,91]. A recent study established T cells derived from T cell receptor (TCR)-engineered iPSCs using a feeder-free protocol [92], with the aim of their utilization in alternative cell-based immunotherapy. Collectively, the generation of HSCs and lineage-specific blood cells from hiPSCs is a complex process of developmental hematopoiesis. It relies on the coordination of multiple factors to achieve efficient production. More extensive studies may fulfill the knowledge in this field and make them more applicable in a clinical setting.

## 4. Genetic Disease Modeling with Patient-Specific iPSCs

**Figure 3:**
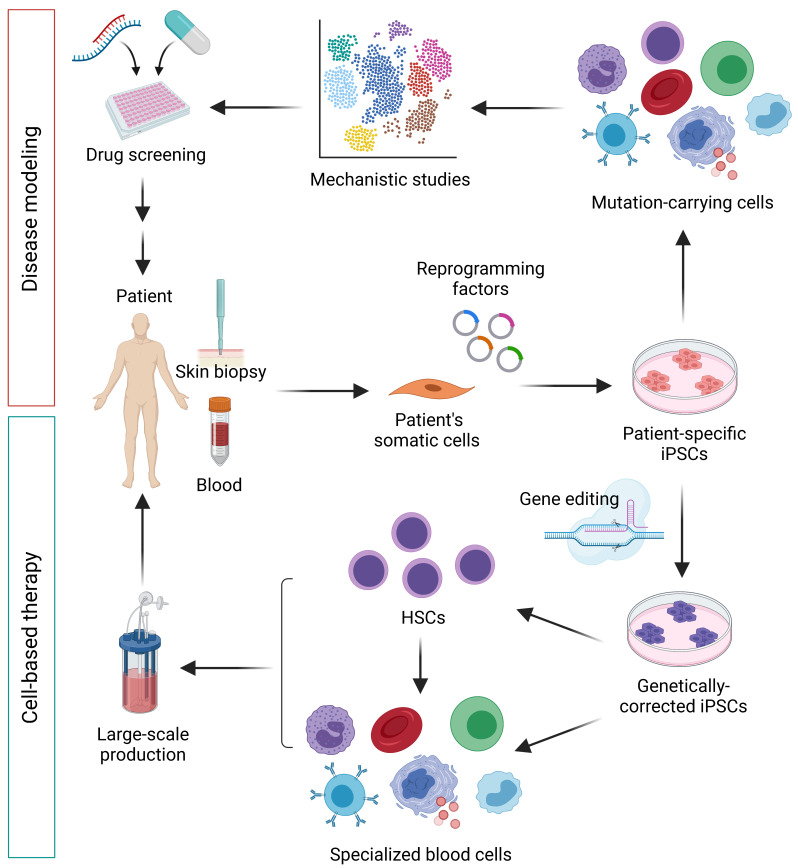
Potential applications of hiPSCs for disease modeling of genetic hematologic disorders and cell-based therapy. Patient-specific iPSCs are a powerful tool for elucidating disease mechanisms and drug screening. Likewise, gene-editing technology can be employed to correct mutations in patient-specific iPSCs, making them a promising cell source for large-scale cell production for cell-based therapy. iPSC, induced pluripotent stem cell; HSC, hematopoietic stem cell.

The emergence of hiPSC technology has contributed to rapid progress in the study of various human diseases, particularly those that have no previous physiologically relevant experimental models. In the past, animal models, frequently mouse models, human primary cells, and immortalized cell lines represented standard experimental tools in basic research to delineate disease pathophysiology, as well as in pharmaceutical research and the development of new drugs. However, mouse models do not always fully recapitulate human phenotypes and biological responses due to species differences, and cell culture models are limited to certain diseases, mainly due to insufficient expandable and/or difficult-to-access cell sources, e.g., cardiomyocytes, neuronal cells, pancreatic cells, and hepatocytes. The use of hiPSCs has solved these hurdles, and due to their property of being able to be maintained without limit in cultures, and their ability to differentiate into virtually any cell type, they have gained much attention in the modeling of diseases with complex genetic defects. Furthermore, hiPSCs can be directly generated from the reprogramming of patients’ somatic cells, allowing a patient’s genetic identity to be preserved in hiPSCs. To date, various cell types differentiated from the patient-specific iPSCs have been shown to recapitulate the disease phenotypes under appropriate conditions, reflecting the correlation of genotypes to cellular phenotypes. In the case of acquired genetic disorders, where disease-causing mutations are only presented in specific tissues, hiPSCs can be established from those normal somatic cells in parallel, thereby providing an opportunity to create normal hiPSCs from the same patient to be used as an isogeneic control. The combination with recent gene-editing techniques, such as RNA-guided clustered regularly interspaced short palindromic repeat (CRISPR)/Cas9), further increases the value of hiPSCs in the modeling of genetic diseases, as well as in clinical cell-based therapy (Figure 3). In this section, we summarize the example studies using hiPSCs for genetic disease modeling for various hereditary and acquired hematologic disorders (Table 1), discuss the major concepts, and demonstrate their potential applications in drug discovery.

### 4.1. Hereditary Hematologic Disorders

Significantly, hiPSCs have been widely used in the modeling of hereditary hematologic disorders in which disease-associated mutations can be passed from the affected individual to the next generation, also known as inherited disorders. The most common and well-known hereditary hematologic disorders are hemoglobinopathies that are caused by mutations in the globin genes. For example, β-thalassemia is characterized by the mutations in β-globin gene (*HBB*), resulting in a reduction in or an absence of β-globin expression. It has been shown that the hiPSC-derived erythroid cells from patients with various forms of β-thalassemia displayed low levels of the *HBB* gene and hemoglobin protein expressions. After genetic modifications, i.e., by homologous recombination or an engineered nucleases-based system, such as zinc-finger nucleases (ZFNs), transcription activator-like effector nucleases (TALENs), and CRISPR/Cas9, corrected iPSCs could be differentiated into erythroid cells with improved β-globin production [93,94,95,96,97,98,99,102,143]. Hemoglobin E (HbE)/β-thalassemia represents the major genotype of those patients affected with severe β-thalassemia worldwide and is the most common form of β-thalassemia found in adults in Southeast Asia. In Thailand alone, at least 3250 new cases of HbE/β-thalassemia are expected annually (1 in 180 births). In HbE/β-thalassemia, one allele (β^0^) produces no β-globin chain, and the other allele (β^E^) produces an HbE globin chain resulting from nucleotide substitution at codon 26 (GAG → AAG, glutamic acid to lysine). Our group used the CRISPR/Cas9 system and a single-stranded DNA oligonucleotide (ssODN) donor template to efficiently correct the HbE mutation in HbE/β-thalassemia patient-derived hiPSCs in one step (2.9% homology-directed repair (HDR)). The corrected iPSCs, which are β-thalassemia heterozygotes, are capable of differentiation into CD34^+^CD43^+^ HSPCs and subsequent erythroid cells that express normal *HBB* and β-globin [93]. The feasibility of genetic correction makes these hiPSCs more attractive for cell therapy by using corrected hiPSC-derived HSPCs for autologous transplantation in the future. Notably, the state of iPSCs has been demonstrated to affect gene correction efficiency—the naïve iPSC reprogrammed from patient with β-thalassemia (41/42, -TCTT deletion) appeared to have higher gene-editing efficiency than those conventional primed iPSCs [144]. 

Another type of hereditary disorder is hemolytic anemia. In this case, patients suffer from RBC destruction before the end of the cells’ normal life span of about 120 days, and hence the patients require blood transfusion or other supportive treatments. A previous study showed that erythroid cells derived from patients’ iPSCs with pyruvate kinase deficiency (PKD), a rare metabolic blood disease caused by the mutation in *PKLR* gene, exhibited energetic imbalances such as decrease in adenosine triphosphate (ATP) levels and metabolites in the glycolysis pathway; the study also showed that the disease phenotypes were restored after knocking in the *PKLR* gene via TALEN-mediated homologous recombination [103]. Hereditary hemolytic anemia could also be caused by mutations in Krüppel-like factor 1 (*KLF1*), a transcription factor controlling almost all aspects of erythropoiesis, although mutations in *KLF1* can vary and lead to different phenotypes ranging from mild to severe phenotypes. A recent study demonstrated disease modeling using hiPSCs derived from a patient with type IV congenital dyserythropoietic anemia (CDA), a rare and severe disease caused by a heterozygous mutation on the second zinc finger of *KLF1* (c.973G > A, p.E325K). The CDA-iPSCs-derived erythroid cells displayed multinucleated morphology, a lack of CD44 expression, and KLF1 target gene dysregulation. Moreover, this study suggested the underlying mechanisms of the p.E325K mutation, which involve the disruption of cell cycle regulators, the cell membrane, and enzyme abnormalities [104]. Recently, our group has generated an hiPSC line from a pediatric patient with severe hemolytic anemia carrying compound heterozygote mutations in *KLF1* (G176RfsX179 and A298P) [145]. Subsequently, the hiPSCs were differentiated toward the erythroid cells. The KLF1-iPSC-derived erythroid cells exhibited relatively low proliferation, poor differentiation, decreased erythroid related-gene expression, and cell-cycle dysregulation when compared to normal hiPSCs, the phenotypes of which could be partially restored after CRISPR/Cas9 gene editing of the G176RfsX179 mutation. 

In addition, hiPSCs have been utilized to model immune system disorders, including severe combined immunodeficiency (SCID) and chronic granulomatous disease (CGD). SCID is a rare disease classified as primary immunodeficiency caused by mutations in multiple genes, e.g., *IL2RG*, *JAK3*, *ADA*, and *RAG1/RAG2*, which are involved in the development of functional T and B cells in the immune system. Several hiPSC modelings of SCID from different mutations have been generated. Previous studies showed that T cells derived from SCID patients with *JAK3* and *IL2RG* mutations exhibited blockage in early T cell development or were unable to produce functional lymphocytes. These defective phenotypes were rescued after gene correction [118,119]. Moreover, recent studies used an SCID-iPSC model to investigate the stage of T cell development and T cell receptor (TCR) rearrangement [89,120]. CGD, which is caused by mutations in the *CYBB* gene encoding NADPH oxidase that is associated with reactive oxygen species (ROS) production in phagocytes, resulted in recurrent infections. The hiPSCs derived from patients with CGD demonstrated the disease-relevant phenotypes resembling those in the patients—that is, the lack of ROS production in CGD-iPSC-derived neutrophils or macrophages [110,111]. Importantly, the defective phenotypes were restored after correction of disease mutations in CGD-iPSCs using different approaches [112,113,114,115,116]. A recent study employed the CRISPR/Cas9 system to first generate patient-like hiPSCs by introducing the p47-ΔGT mutation into normal hiPSCs and subsequently mediating gene editing of the introduced mutation. Granulocytes differentiated from such corrected hiPSCs could restore gene expression and express functional NADPH oxidase activity with bacteria-killing capacity [117]. 

### 4.2. Acquired Hematologic Disorders

Acquired hematologic disorders are not inherited, and disease-causing mutations are acquired and restricted only in certain hematopoietic cells, resulting in cell heterogeneity in patients. Disease-specific hiPSCs can be then generated from the abnormal clones, while normal hiPSCs that do not carry genetic mutations can be generated from unaffected tissues to serve as germ-line controls in experiments or can be used as autologous cells for therapeutic purposes. Hematologic malignancies are a good example of acquired hematologic disorders, which contain very heterogeneous subpopulations. Samples collected from the patients’ bone marrow or PBMCs could be a mixture of normal cells and premalignant and malignant clones, depending on the disease progression, remission, and administered therapies. It is believed that generated hiPSCs from different clones can be used to study clonal evolution that is a critical event in cancer development. However, efforts to develop hiPSC models of hematologic malignancies are thus far limited to myeloid malignancies, namely, myeloproliferative neoplasms (MPN), myelodysplastic syndromes (MDS), MDS/MPN overlap syndromes, and acute myeloid leukemia (AML), mainly due to the difficulty of generating lymphoid lineages from hiPSCs. 

MDS, earlier described as preleukemia or smoldering leukemia, is a bone marrow failure syndrome characterized by impaired hematopoiesis. Previously, hiPSCs were derived from the hematopoietic cells of MDS patients with chromosomal 7q deletions (del7q) in parallel with isogenic normal hiPSCs from hematopoietic cells. del7q-iPSCs displayed hematopoietic defect phenotypes after differentiation, which could be rescued after chr7q dosage correction. This system was extended to identify the candidate disease-specific haplo-insufficient genes, such as *EZH2* and *LUC7L2*, which might mediate the hematopoietic defects in MDS via phenotype-rescue screening [134]. To explore the disease stage transition, a panel of hiPSCs capturing distinct disease stages from preleukemia, low-risk MDS, high-risk MDS, and secondary AML, was created. Upon differentiation, the disease-stage-specific iPSCs could recapitulate the graded severity of cellular phenotypes and/or stage specificity observed in the patients. This study also demonstrated the use of a CRISPR/Cas9 system to induce both disease progression and reversal via genetic correction or introduction of mutations, which may aid in the mechanistic understanding of myeloid transformation and drug testing for stage-specific therapeutic interventions [135].

Another study also used an hiPSC model of MDS derived from single premalignant cells with a partial complement of mutations in an investigation of acquired genetic changes and their functional consequences on disease progression from MDS-iPSC subclones. Using this approach, the study was able to identify the events that occurred during leukemic progression, for example, *SF3B1* mutations, in concert with epigenetic mutations that could disturb mitochondrial function, leading to damaged mitochondria and resulting in apoptosis and impaired erythropoiesis [146]. A recent study developed a model of clonal evolution of AML by sequentially introducing the associated mutations, i.e., *ASXL1*, *SRSF2*, and *NRAS*, using CRISPR/Cas9 technology in hiPSCs, and characterized the transcriptional program and signaling pathways upon disease development using transcriptomic and chromatin analyses. This study also suggested a novel therapeutic approach that targets early AML via an inhibition of inflammatory signaling, which is an early and persistent event in leukemogenesis [147]. 

Additionally, hiPSCs from AML patients with *MLL* rearrangements have been successfully established, in which the mutations were preserved in the derived AML-iPSCs, while leukemic DNA methylation and the gene-expression profile were reset after reprogramming. Upon hematopoietic differentiation, the leukemic properties were reactivated as indicated by an aberrant myeloid-restricted phenotype and aggressive myeloid leukemia after transplantation into immunodeficient mice. In addition, distinct AML-iPSC subclones were used to identify target therapy at a specific stage of AML [136]. hiPSC models of chronic myeloid leukemia (CML) have been developed displaying disease phenotypes such as impaired hematopoietic differentiation, resistance to tyrosine kinase inhibitors (TKIs), and heterogeneity of the clones [137]. These models were used to gain more extensive insight into the mechanisms of TKI resistance in CML stem cells and were evaluated as therapeutic candidates that could be used to prevent disease recurrence [138,139,140,141]. 

Polycythemia vera (PV) and essential thrombocytothemia (ET) are BCR-ABL1 negative MPNs, involving an increased red cell mass and clonal platelet overproduction, respectively. *JAK2* is the most common target of driver mutations with frequencies of approximately 98% in PV and 50–60% in ET. Previous studies generated iPSCs from cells of MPN patients carrying the JAK2V167F mutation, usually detectable in both PV and ET, and *JAK2* exon12 mutations found in PV, and subsequently differentiated them toward a hematopoietic lineage. JAK2exon12-iPSCs, but not JAK2V617F-iPSCs, exhibited an increased erythropoiesis resembling the pathophysiology in PV patients. JAK2V617F-iPSCs did not exhibit a significant increase in erythroid cell proliferation or differentiation, most likely because the studied JAK2V617F expression was more consistent with the heterozygous JAK2V617F mutation in ET patients, where the endogenous *JAK2* gene was still present [148,149]. The utilization of different types of *JAK2* mutations in patient-derived iPSCs or generated mutant iPSCs for the discovery of pathogenesis and candidate therapy for MPNs has been demonstrated [149,150]. Additionally, hiPSCs derived from patients with the calreticulin (*CALR*) gene mutation, which is usually found in ET and primary myelofibrosis patients, could reflect disease phenotypes by representing megakaryopoiesis and prominent colony-forming unit megakaryocytes (CFU-MK) [151,152].

## 5. Patient-Specific iPSCs for Drug Screening

Besides applications in studying disease mechanisms, patient-specific iPSCs have contributed greatly to therapeutic development due to their limitless availability and capacity to differentiate into relevant cell types while reflecting human disease phenotypes. Compared to conventional animal-based testing and cell-line-based compound screening, the hiPSC-based platform is a more comprehensive, powerful tool for predicting the safety and efficacy of drug responses in the human setting, thus saving time and costs in the development process. Thus far, various patient-specific iPSC models for drug screening for patients with hematologic disorders have been demonstrated. 

An early example platform for drug screening for hereditary blood diseases is the use of hiPSCs derived from X-linked CGD patients as a robust model for testing enzyme replacement therapy. The researchers utilized recombinant NOX2/p22phox proteoliposomes containing an active cytochrome b558, the membrane component of NADPH oxidase complex, and delivered it to CGD-iPSC-derived macrophages. After treatment with specific liposomes, NADPH oxidase activity was restored without toxicity, making it a potential approach for future treatment of pulmonary infection in CGD patients [153]. For Fanconi anemia (FA), a congenital disorder characterized by bone marrow failure, FA-iPSCs were used for drug screening of several compounds known to improve FA phenotypes, such as resveratrol (Sirt1 activator), danazol (synthetic androgen), and doramapimod (p38 MAPK inhibitor), based on the evaluation of the effects on hematopoietic differentiation. Of those compounds, doramapimod and tremulacin (anti-inflammatory) significantly improved the production of CD34^+^CD43^+^ HSPCs upon FA-HSC differentiation through a mechanism that most likely involved the suppression of proinflammatory cytokines, including IFNγ, TNF, and IL-6, at the transcriptional level [154]. Alternatively, for Schwachman–Diamond syndrome (SDS), a bone marrow failure syndrome with propensity to develop MDS and AML, researchers first used the iPSC model to identify the underlying mechanisms and candidate therapeutic target before proposing potential therapeutics. Both iPSC-derived from SDS patients and engineered del(7q) iPSCs suggested the TGF-β pathway as the candidate target. After adding small molecule SD208, an inhibitor of TGF-β receptor I kinase, into the culture, the hematopoiesis of SDS-iPSCs was rescued, as shown by an increase in both the number and size of erythroid and myeloid colonies. However, similar results could not be observed for the engineered del(7q) iPSCs, thereby suggesting that hiPSCs can be used to study the distinct contributions of somatic alterations [155].

A recent study performed chemical screening on iPSCs derived from a Diamond–Blackfan anemia (DBA) patient and identified that SMER28, a small molecule modulator of autophagy, has the potential to cure DBA. SMER28 was shown to stimulate erythropoiesis in DBA-iPSCs by promoting autophagy in erythroid progenitors through autophagy factor ATG5 and also upregulated the expression of globin genes [132]. Another group also used a DBA-iPSC model and identified a new therapy, namely, eltrombopag (EPAG), a Food and Drug Administration (FDA)-approved synthetic small-molecule mimetic of TPO. EPAG partially rescued erythropoiesis by mediating intracellular iron restriction in DBA-iPSC-derived erythroid cells, which was in common with the effects of deferasirox (DFX), a clinically licensed iron chelator, on DBA-iPSCs [156].

The discovery of therapeutic drugs has also been successful in hiPSCs derived from patients with acquired blood disorders. For example, a drug-testing system was established from hiPSCs derived from a patient with chronic myelomonocytic leukemia (CMML), a clonal HSC disorder with overlapping features of MDS and MPN. In this study, the researchers used MEK inhibitor PD0325901 and Ras inhibitor salirasib to treat CMML-iPSC-derived CD34^+^CD43^+^ HPCs and observed its capacity to suppress colony formation [157]. Moreover, phenotypic screening using liposomal clodronate, which has been previously shown to deplete monocytes in a monocyte/macrophage system, was tested under this setting. The results showed that liposomal clodronate decreased the number of colonies and reduced serial replating capacity in CMML-iPSC-derived HPCs, indicating a potential drug reposition of this clinically used osteoporosis drug. The findings also recommend hiPSCs as a rational model for the identification of drug candidates for repositioning.

Drug testing for disease-stage-specific therapeutic interventions could be performed using hiPSC models. In a myeloid malignancy study, a panel of disease-stage-specific iPSCs for modeling MDS and secondary AML was established, as mentioned above. The researchers applied 5-Azacytidine (5-AzaC), a hypomethylating agent used as first-line therapy of MDS, to different MDS-iPSCs-derived HPCs and observed its selective effect on stage-specific iPSC lines, i.e., high-risk MDS was the most responsive. Moreover, they also reported that HPCs derived from a less- and a more-advanced disease stage of MDS/AML-iPSCs displayed different responses to rigosertib, a small molecule inhibitor of RAS signaling currently undergoing clinical trials for high-risk MDS, with the KRAS-mutated line being more responsive to the treatment [134]. Another study identified drug sensitivity on a CRISPR/Cas9-mediated MDS-iPSC model. Treatment of engineered SRSF2-mutant iPSC-derived HPCs with splicing inhibitor E7107 and splicing-modulating drugs Cpd-1, Cpd-2, and Cpd-3, which are small molecule inhibitors of CDC-like kinases and serine-arginine protein kinases, exhibited a selective growth-inhibitory effect. Further screening from a library of 2000 compounds containing FDA-approved drugs, natural products, and other bioactive compounds found that niflumic acid was identified to have selective inhibition on the growth of del(7q) iPSC-derived HPCs [158].

## 6. iPSCs and Therapeutic Applications

The promise of hiPSCs lies in their far-reaching potential to serve as a starting material by which to develop a range of therapeutic cells from their capability to multiply indefinitely and the ability to generate virtually any cell types upon differentiation in vitro. The feasibility of performing genetic manipulation further advances the therapeutic applications of hiPSCs for genetic disorders. One of the major concerns for clinical-grade iPSCs is the comparability of lines derived from different individuals and in different facilities. Previously, guidelines on critical quality attributes (CQAs) and minimum testing requirements for clinical-grade iPSC lines have been outlined: (i) identity by single tandem repeat (STR) genotyping; (ii) microbiological sterility; (iii) genetic fidelity and stability by testing for residual reprogramming vectors and karyotyping; (iv) viability; (v) characterization of markers from standard hPSC panel; and (vi) potency, which is the qualitative measure of the biological activity of the cells [159]. In this section, we discuss the uses of hiPSCs as an alternative cell source for cell-based regenerative therapy for hematologic disorders and for cell-based cancer immunotherapy in both solid tumors and hematologic malignancies.

### 6.1. Cell-Based Therapy

In the past, treatment options for inherited or acquired hematologic disorders mostly relied on supportive treatment, including blood transfusion, medication, surgery, and chemotherapy, aiming to relieve signs and symptoms associated with the disorder. However, these approaches are unable to completely eradicate the particular genetic causes in the patients. Hematopoietic stem cell transplantation (HSCT) has become the standard of care for many patients with malignant and non-malignant hematologic disorders, as it can reestablish lifelong blood cell production, replacing affected blood cells and allowing for a definitive cure for the disorders. Still, the major challenge of HSCT is the availability of appropriate and sufficient donor cells for robust hematopoietic repopulation. HSCT is classified into allogeneic and autologous HSCT, according to whether the cell source is from healthy donors or the patients’ own stem cells, respectively. The advantages of autologous over allogeneic HSCT are the lower risk of life-threatening complications, such as graft-versus-host disease (GvHD), graft failure and infections, and the relatively good tolerance in elder patients. However, autologous HSCT may require gene modification prior to transplantation. On the other hand, allogeneic HSCT may be limited by the availability of compatible donors and in the case of hematologic malignancies, autografts may be contaminated with tumor cells and patients will not benefit from the graft-versus-tumor effect observed after allogeneic HSCT. HSCs can be harvested from bone marrow aspiration, apheresis of stimulating factor-mobilized peripheral blood (the most common source), and umbilical cord blood. Although umbilical cord blood is rich in HSCs and enables greater tolerance to human leukocyte antigen (HLA) mismatching than other sources, cell numbers remain insufficient in large adult hosts; hence, it requires ex vivo expansion [160]. Considering the feasibility for gene modification and generation of HSCs, hiPSCs are a good candidate cell source for autologous HSCT. As mentioned earlier, disease-specific iPSCs can be derived from patient’s somatic cells, and the disease-causing mutation can be corrected prior to HSC differentiation. In the case of acquired hematologic disease, healthy clones of hiPSCs derived from the patient can be directly used to produce autologous HSCs, which are of less concern for immune rejection. Our group generated disease-free iPSCs from the fibroblasts of a patient with paroxysmal nocturnal hemoglobinuria (PNH), whose granulocytes and red blood cells consist of a minor normal population and major population with decreased surface CD55 and CD59 [161]. After hematopoietic induction, the differentiated cells from PNH fibroblast-iPSCs expressed early hematopoietic markers (CD34 and CD43) with normal CD55 and CD59 expression similar to those of their parental cells, suggesting that PNH fibroblast-iPSCs can be a potential source of HSCs for autologous transplantation to cure PNH patients. Notably, the first clinical trial of hiPSC-based cell therapy was in patients with neovascular age-related macular degeneration (AMD) [162], where retinal pigment epithelial (RPE) cells were derived from patients’ iPSCs and transplanted back as an RPE cell sheet under the retina (#UMIN000011929). After over 2 years of transplantation, the safety and efficacy were confirmed with no sign of immune rejection. Recently, another clinical trial was undertaken using an hiPSC-immortalized megakaryocyte cell line (iMKCL) mediated with c-MYC, BMI1, and BCL-XL as a source for platelet production for a patient with thrombocytopenia from aplastic anemia [163]. Collectively, hiPSCs hold great promise for both autologous HSC and iPSC-derived blood cells for cell replacement therapy.

Notably, hiPSCs are being preferentially considered for allogeneic transplantation due to the fact that personalized hiPSC-based cell products require high cost, a complex cell facility, and are time consuming, making them impractical for acute conditions. One of the current strategies to circumvent immune rejection in allogeneic hiPSC applications is by generating hiPSCs from various HLA homozygous donors, which involves matching of the worldwide population at the major loci and stocking these cells in biobanks. However, this approach is very challenging for donor eligibility as it requires a large number of HLA-homozygous iPSCs to maximize the coverage [164,165]. Another approach is to adopt genome-editing technologies to generate hiPSCs with enhanced immune compatibility to avoid immune rejection in allogeneic recipients caused by HLA mismatching. For example, a CRISPR/Cas9 system was recently used to delete HLA-A and HLA-B biallelically, while retaining a single haplotype of HLA-C and non-canonical HLA-E, -F, and -G of hiPSCs, the strategy of which was shown to be effective at suppressing CD8^+^ T and NK cell activities [166]. Alternatively, universal hiPSCs may be generated by the disruption of β2-microglobulin (B2M) and class II transcription activator (CIITA) [167,168,169,170,171], causing major histocompatibility complex (MHC) class I and II inactivation, respectively, together with the introducing of HLA-E/B2M fusion or the CD47 transgene [169]. Notably, depletion of B2M alone would activate a response by NK cells as HLA class I molecules serve as an NK inhibitory signal. Collectively, these approaches provide hypoimmunogenic hiPSCs that can be further differentiated into various cell types without immunosuppression. 

The potential applications of hiPSCs have also been demonstrated in combination with gene therapy for various hematologic disorders. The first proof-of-principle study was performed in a humanized, sickle cell anemia mouse model, where sickle cell manifestations can be rescued after transplantation of corrected hematopoietic progenitors derived from autologous iPSCs [172]. Further, the attempts to use hiPSC-based gene therapy have been demonstrated in various forms of SCID (JAK3-SCID, ADA-SCID, X1-SCID), in which corrected hiPSC-derived HSCs exhibited normal T cell development and could yield mature NK cells [118,119,173] and in CGD [110,112,115], as mentioned above. In addition, the feasibility of hiPSC-based gene and cell therapies has been demonstrated in hemophilia A, a bleeding disorder caused by mutations in the *F8* gene. The researchers generated corrected hiPSCs using a lentiviral vector carrying the FVIII transgene and differentiated them into functional endothelial cell (ECs) followed by transplantation into immunodeficient mice. The hiPSC-derived ECs could produce functional FVIII and rescued the hemophilic phenotype after transplantation [127]. For hemophilia B, CRISPR/Cas9-mediated *F9* gene correction was performed in patients’ iPSCs prior to their differentiation into hepatocyte-like cells (HLCs) and transplantation into a hemophilic mice model. The presence of FIX in corrected, transplanted hepatocytes was observed 6–9 months after transplantation [128]. 

### 6.2. Cell-Based Cancer Immunotherapy

Cancer immunotherapy is a transformative treatment for cancers that provides hope to patients with relapsed and refractory diseases, who are not responsive to conventional therapies. The concept of cancer immunotherapy arose from the tumor immune escape, one of the hallmarks of cancers, aiming to restore the host immune defense to keep cancer permanently at bay. Chimeric antigen receptors (CARs) are engineered transmembrane receptors that engage immune cells towards particular tumor-associated antigens. The clinical success stories of CAR-T cell therapy targeting B-cell malignancies—with four US FDA-approved autologous CAR-T cell products for the treatment of relapsed/refractory diffused large B-cell lymphoma (DLBCL), acute lymphoblastic leukemia (ALL), and mantle cell lymphoma—have made cellular immunotherapy an attractive field [174,175]. The number of CAR technology-related clinical trials has been increasing exponentially. Amidst this enthusiasm, several challenges to cellular immunotherapy from a technical perspective are: (i) the limited number of patients’ immune cells obtained and their exhaustion; (ii) the difficulty in engineering primary cells and their fragility after the engineering process; (iii) the limited ability of primary immune cells to proliferate, which hinders clonal selection; and (iv) the personalized, autologous nature of certain immune cellular products to avoid the risk of deleterious GvHD [176]. Therefore, hiPSCs, which can easily be genetically modified and maintained practically indefinitely in culture and differentiated into any immune cells upon induction, are an ideal alternative cell source for developing next generation CAR-related immune cell therapy. 

One important drawback of the current CAR-T cell therapy is its personalized, autologous form that is made for individual patients and cannot be prepared as off-the-shelf products. Hence, its noticeable limitation is associated with the manufacturing process, which is costly and laborious, increasing the risk of production failure in clinical settings, especially in those with a limited number of healthy T cells, rendering these CAR-T cells unsuitable for patients with rapidly progressing disease [177]. A previous study provided evidence that genetic engineering of hiPSCs with second-generation CARs would be an efficient strategy to generate functional and expandable CAR-T cells. To this end, hiPSCs were generated by reprogramming peripheral blood T lymphocytes from healthy donors and were subsequently transduced with second-generation anti-CD19 CAR and differentiated into T cells using the O9P9-DL cell lines. The differentiated anti-CD19 CAR-T cells displayed canonical features of T cell function and specificity towards CD19, suggesting the feasibility of generating CAR-T cells from hiPSCs [178]. Numerous efforts have been proposed to adopt genome-editing technologies to generate hiPSCs with enhanced immune compatibility, as mentioned above. Hence, subsequent incorporation of CARs into those hypoimmunogenic hiPSCs may enable the production of off-the-shelf, allogeneic CAR-T cells. 

NK cells are part of the first line of defense and are rapidly activated without prior sensitization to protect the body against foreign materials and abnormal cells, including tumor cells. Allogeneic NK cells do not carry the risk of inducing GvHD, which is frequently associated with allogeneic CAR-T cells; therefore, they can be prepared as off-the-shelf products without further modifications [179]. Currently, several phase 1 clinical trials have utilized allogeneic hiPSCs as the source of NK cells for immunotherapy of various solid tumors and hematologic malignancies (NCT03841118; NCT04245722; NCT04106167; NCT04023071; NCT04551885). Of them, anti-CD19 CAR was expressed in hiPSCs together with a cleavable CD16 Fc receptor and an IL-15 receptor fusion prior to the induction of NK differentiation towards CAR-NK cells for the treatment of relapsed/refractory lymphoma and chronic lymphocytic leukemia (CLL) (NCT04245722) [180]. Notably, CAR-NK cells could eliminate tumors not only through the ability of CAR to target specific tumor antigens, but also through antigen-unrestricted killing activity of NK cells themselves, namely, granule-mediated cytotoxicity, cytokine-mediated cytotoxicity, and CD16-mediated antibody-dependent cell-mediated cytotoxicity (ADCC) [181].

In solid tumors, CAR-T cells have shown low success rates, mainly due to the high complexity of the tumor microenvironment containing an immunosuppressive network and physical and metabolic barriers [182,183,184]. Recently, immune cells of myeloid lineage, i.e., monocytes and macrophages, have gained increasing attention due to their potential capability of active accumulation in solid tumors and penetration into the dense stromal tissues surrounding tumors. It is noticeable that allogeneic CAR-macrophages carry a low risk of GvHD and can also be prepared off-the-shelf similar to those of CAR-NK cells [184]. Data from a phase 1 clinical trial of autologous anti-mesothelin CAR-macrophages for ovarian cancer and malignant peritoneal mesothelioma have shown preliminary safety with no treatment-related discontinuations (NCT03608618) [185], giving hope for the applications of novel cellular immunotherapy for solid tumors. The feasibility of the derivation of anti-CD19 CAR-macrophages—with antigen-dependent phagocytosis and antitumor functions in vitro and in vivo—from hiPSCs has recently been shown, although the efficacy and persistency need to be further improved by designing more suitable CARs [186]. 

## 7. Current Challenges and Future Perspectives

With the discovery of hiPSCs over the past decade, rapid progress has been made in biomedical and pharmaceutical research. hiPSCs open up research opportunities as a new platform for disease modeling and therapeutic drug development and screening; additionally, their translation to potential clinical applications, such as cell-based therapy and immunotherapy, has exponentially increased. The advantages of hiPSCs are even more pronounced when combined with other technologies, e.g., genome-editing technology, which helps either recapitulate or correct the disease phenotypes. However, several limitations remain to be further investigated. 

For hiPSC-based disease modeling, patient-specific hiPSCs have been used as representative cells of patients that carry specific mutations, which could display particular disease phenotypes once differentiated in vitro. hiPSCs serve as an alternative to human primary cells that might not be available or easily accessible, and to complement animal models that might not resemble human pathophysiology. Thus far, numerous disease models using hiPSCs have been demonstrated; however, one of the concerns raised is the respective appropriate control—genetic variations by means of gene expression pattern and epigenetic status even in control hiPSCs were reported either from within or between individual donors [187,188]. These background genetic variations have a remarkable impact on the differentiation capacity of hiPSCs, resulting in a large heterogeneity of lineage phenotypes that confounded the interpretation of the data in disease modeling [189,190,191]. To this end, genome-editing technology, i.e., CRISPR/Cas9, was utilized to generate a genetically matched hiPSC line, known as isogenic correction control iPSCs, for use as comparative control cells in the study. Using this approach, variation in genetic background and other confounders would be eliminated. It is important to note that the possibility of potential off-target effects remains challenging. Alternatively, some studies have attempted to convert the primed state of hPSCs into a naïve state in order to overcome the heterogeneity, based on the findings in mouse ESCs. Several approaches have been used to induce a naïve state of hESCs, i.e., by adding a combination of kinase inhibitors, such as MEK, GSK3, ROCK, BRAF, and SRC in the presence of activin and hLIF [192] and overexpression of transcription factors NANOG and KLF2 [193]. However, these data are preliminary and concerns regarding the naïve state, e.g., genetic integrity and loss of imprinting, need to be further clarified.

Considering the ability of hiPSCs to differentiate into disease-relevant cell types and various cell products, it has been shown that certain differentiated cells, with most evidence from blood cells, exhibited immature functional characteristics. Several studies demonstrated that HSPCs differentiated from hiPSCs do not truly resemble bona fide HSCs with long-term repopulation, also known as definitive HSCs. In addition, terminal differentiation of iPSC-derived HSCs toward a specific lineage, such as an erythroid lineage, produced mostly erythroid cells with embryonic or fetal globins, and had a low enucleation rate. These limitations may slow the progress of hiPSC-based disease modeling, cell-based therapy, and drug discovery. To overcome these obstacles, high-efficiency differentiation protocols should be developed. It has been demonstrated that the hematopoietic niche is mainly involved in HSC production. A recent study showed that AGM-associated macrophages played roles in HSC production by providing the microenvironment for definitive EHT and hematopoietic maturation [191], suggesting that appropriate niches are very important for cellular development. A more recent study utilized single-cell transcriptomics to identify novel mechanisms that play a key role in definitive hematopoietic production, and cell cycle regulators, e.g., CDK4/6 and CDK1, have been identified as essential for EHT and hematopoietic differentiation [194]. Therefore, using a hiPSC differentiation system combined with other new technologies may provide more in-depth understanding in human hematopoiesis that could lead to the establishment of efficient differentiation methods/protocols for better production of functional blood cells in the near future. Of note, the use of small molecule inhibitors/enhancers is gaining interest as an attractive method for both reprogramming and improved cell-specific differentiation that are appropriate for clinical applications, due to the ease of use and zero risk of integrating exogenous genetic factors. 

To date, the applications of hiPSCs for clinical purposes such as cell-based therapy are an active area of research. One of the critical issues is potential tumorigenicity, attributable to the residual undifferentiated and/or immature cells derived from hiPSCs existing in the final cell products, which could possibly lead to the emergence of teratomas or tumors after transplantation [195,196,197,198]. Many efforts have been made to overcome these challenges. For example, the establishment of efficient directed differentiation methods; quality control, particularly on the cell purification using positive and/or negative antibody selection; and the use of safety switches that selectively eliminate the tumorigenic cells. It has been shown that suicide genes, i.e., herpes simplex virus type 1 thymidine kinase (*HSVtk*), and drug-inducible safeguard systems could be used to deplete immature proliferating cells or undifferentiated hiPSCs and hiPSC-derived cells after transplantation [199,200], making hiPSC-derived cell therapy safer. Autologous hiPSC transplantation has been considered a promising tool for therapeutic application due to its safer profile of immune responses. However, the major drawbacks of this strategy are the cost of production and the limited availability/accessibility, which is critical for patients with rapidly progressing conditions, as death may occur prior to the cell therapy, e.g., from heart failure or spinal cord injury. These limitations could be potentially overcome by using allogeneic hiPSC transplantation, but only if the issues with immunogenicity have been resolved. Notably, comprehensive quality testing, including contamination tests, such as sterility and viral testing, morphology, HLA and STR analyses to prevent sample mix-ups, pluripotent markers, karyotyping, and genomic analyses to evaluate genomic mutations, are mandatory for clinical-grade hiPSCs.

## 8. Conclusions

In conclusion, hiPSC-based disease modeling, with cells either obtained from patient-specific somatic cells carrying specific mutations or from the introduction of normal iPSCs with specific mutations, offers an exclusive and convenient means to model disease phenotypes that also allows studies to track disease progression in the case of acquired genetic disorders. Although several concerns still limit the validity of hiPSC-based disease modeling, most of these limitations can be overcome by following some general guidelines or by mindfully interpreting the data. For example, the heterogeneity of hiPSCs and their differentiated progeny among different individuals can be overcome by using an isogenic, unaffected control whenever possible. Higher numbers of clones and better standardization of efficient differentiation protocols are necessary for reduced variability and increased reproducibility. Although the functional immaturity of the iPSC-derived blood cells remains an important issue to be addressed, hiPSC-based disease modeling provides a greater understanding of the pathophysiology, particularly early events that could potentially lead to the identification of novel therapeutic targets, and it is a useful tool for high-throughput screening of novel drugs. hiPSCs can also be viewed as a game changer for cell-based therapy. hiPSCs hold great promise for both autologous HSCs and iPSC-derived blood cells/products. However, an increasing trend has been the clinical applications of hypoimmunogenic, allogeneic iPSC-based cell therapy, attributable to the laborious production and time delay of personalized cells. Recently, CAR-T cell therapy has become a new hope for cancer treatment, leading to the rapid growth in cellular immunotherapy. hiPSCs have been listed as an ideal alternative cell source for cancer immunotherapy, especially when certain allogeneic immune cells, such as CAR-NK cells and CAR-macrophages, carry a low risk of immune rejection and GvHD. Overall, hiPSCs greatly benefit biomedical and biopharmaceutical research and offer great opportunities for future regenerative medicine.

## Figures and Tables

**Table 1:** List of the selected studies using hiPSCs for genetic disease modeling for hereditary and acquired hematologic disorders.

Disorder	Genotype	Cell Source	Phenotypes (vs. Normal hiPSCs)	Genetic Modifications	Phenotypic Rescue (vs. Disease-Specific hiPSCs)	References
**Hereditary Hematologic Disorders**						
β-thalassemia major	HbE/β-thalassemia (β^E^/β^0^ (β41/42))	Fibroblasts	HbE/β-thalassemia iPSCs produced lower hematopoietic progenitor cells and erythroid cells	CRISPR/Cas9-mediated HbE correction	Restored the number of hematopoietic progenitor cells and erythroid cells	[93]
	Homozygous β-thalassemia (β^0^/β^0^ (β41/42))	PBMCs, fibroblasts	β41/42-thalassemia iPSCs displayed lower differentiation efficiency and produced erythrocytes with absence of *HBB* gene and protein expression	CRISPR/Cas9-, HR-mediated *HBB* correction,	Restored *HBB* gene and protein expression in the corrected iPSC-derived erythrocytes	[94,95,96,97]
	Homozygous β-thalassemia (β^+^/β^+^ (IVS2-654))	Fibroblasts, amniotic fluid	IVS2-654 thalassemia iPSC-derived erythrocytes lacked *HBB* gene and protein expression	TALEN-, ZFN-, or CRISPR/Cas9-mediated *HBB* correction	Restored *HBB* gene and protein expression in the corrected iPSC-derived erythrocytes	[98,99,100,101]
α-thalassemia	Homozygous α-thalassemia major (− −/− −)	Fibroblasts	Homozygous α-thalassemia iPSC-derived erythrocytes expressed no α-globin chains	ZFN-mediated *HBA1* correction	Improved globin chain imbalance in the corrected iPSC-derived erythrocytes	[102]
Hemolytic anemia	Heterozygous *PKLR* (359C > T) and (1168G > A) and homozygous *PKLR* (IVS9(+1)G > C)	PB	PKD-iPSC-derived erythroid cells displayed the energetic imbalance	TALEN-mediated *PKLR* correction	Recovered energetic balance in the corrected iPSC-derived erythroid cells	[103]
	Heterozygous *KLF1* (c.973G > A, p.E325K)	PBMCs	CDA-iPSC-derived erythroid cells displayed multinucleated morphology, absence of CD44, dysregulation of target gene and cell cycle regulator genes	N/A	N/A	[104]
SCD	Homozygous (β^S^/β^S^)	BM, PBMCs, Fibroblasts	N/A	ZFN-, TALEN-, CRISPR/Cas9-mediated *HBB* correction	Restored *HBB* gene and protein expression in corrected iPSC-derived erythrocytes	[105,106,107,108,109]
CGD	Homozygous and heterozygous *CYBB* mutations	BM, PB CD34^+^ cells, fibroblasts, keratinocytes	CGD-iPSC-derived neutrophils and macrophages lacked ROS production	ZFN-, CRISPR/Cas9-, HR-, TALEN-mediated *CYBB* correction	Restored *CYBB* gene expression, functional NADPH oxidase activity, and antimicrobial activity in corrected iPSC-derived neutrophils or macrophages	[110,111,112,113,114,115,116,117]
SCID	X-SCID (*IL-2Rg* 468 + 3A > C)	BM	SCID-X1-iPSCs could not differentiate into functional lymphocytes	TALEN-mediated *IL2RG* correction	Recovered the production of mature NK cells and T cell precursors differentiated from corrected SCID-X1-iPSCs	[118]
	JAK3-SCID Homozygous (*JAK3* 613C > T)	Keratinocytes	*JAK3* mutant iPSCs exhibited blockage in early T cell development	TALEN-mediated *JAK3* correction	Restored normal T cell development in corrected *JAK3* mutant iPSCs	[119]
	RAG1-SCID Homozygous and compound heterozygous *RAG1* mutations	Fibroblasts	*RAG1* mutant iPSCs displayed blockage in early T cell development and TCR re-arrangements	N/A	N/A	[89]
	RAG2-SCID Homozygous *RAG2* (p.R148X)	Fibroblasts	*RAG2* mutant iPSCs displayed blockage in early T cell development and TCR re-arrangements	HR-mediated *RAG2* correction	Restored normal T cell development and TCR rearrangements in corrected *RAG2* mutant iPSCs	[120]
WAS	*WASP* (c.1507T > A) and (c.55C > T)	Fibroblasts	WAS-iPSCs exhibited defects in platelet production	Overexpression of *WASP* using lentiviral vector	Improved proplatelet structure and increased the platelet size in overexpressed WAS-iPSCs	[121]
	*WASP* 1305 insG	Fibroblasts	WAS-iPSCs exhibited deficient T lymphopoiesis and NK cell differentiation and function	ZFN-mediated *WASP* correction	Restored T and NK cell differentiation and function in corrected WAS-iPSCs	[122]
Hemophilia A	*F8* mutations	Fibroblasts, epithelial cells, PB CD34^+^ cells	HA-iPSCs-derived endothelial cells lacked *F8* gene expression, secretory protein, and activity	TALEN-, CRISPR/Cas9-, lentiviral vector-mediated *F8* correction	Restored F8 transcript, protein secretion, and activity in corrected HA-iPSCs both in vitro and in vivo	[123,124,125,126,127]
Hemophilia B	*F9* mutations	PBMCs	HB-iPSCs-derived hepatocyte-like cells could not secrete coagulation factor FIX	CRISPR/Cas9-mediated *F9* correction	Restored F9 transcript, protein secretion, and activity in corrected HB-iPSCs both in vitro and in vivo	[128,129,130]
DBA	*RPS19* and *RPL5* mutations	Fibroblasts	DBA-iPSCs exhibited ribosomal defects, impaired erythropoiesis	ZFN-, CRISPR/Cas9-mediated *RPS19* or *RPL5* correction	Rescue of ribosomal defects and erythropoiesis in corrected DBA-iPSCs	[131,132,133]
**Acquired Hematologic Disorders**						
MDS	del(7q)	BM, PBMCs	MDS-iPSCs exhibited impaired hematopoietic differentiation, clonogenic capacity, cell growth, and viability	Spontaneous dosage chr7q correction, CRISPR/Cas9-mediated gene correction	Restored hematopoietic differentiation in corrected MDS-iPSCs	[134,135]
AML	*MLL* rearrangement	Primary AML cells	AML-iPSCs exhibited leukemic behavior and methylation patterns upon hematopoietic differentiation	N/A	N/A	[136]
CML	BCR/ABL	PBMCs, BM	CML-iPSCs resistant to tyrosine kinase inhibitor (TKI) and reduced hematopoietic differentiation	N/A	N/A	[137,138,139,140,141]
PNH	*PIGA* mutations	Fibroblasts	*PIGA*-iPSCs were unable to produce hematopoietic cells or mesodermal cells expressing KDR/VEGFR2 and CD56 markers	N/A	N/A	[142]

Abbreviations: iPSC, induced pluripotent stem cell; CRISPR, clustered regularly interspaced short palindromic repeat; PBMC, peripheral blood mononuclear cell; HR, homologous recombination; ZFN, zinc-finger nuclease; PB, peripheral blood; PKD, pyruvate kinase deficiency; TALEN, transcription activator-like effector nuclease; CDA, congenital dyserythropoietic anemia; N/A, not available; SCD, sickle cell disease; BM, bone marrow; CGD, chronic granulomatous disease; ROS, reactive oxygen species; SCID, severe combined immunodeficiency; TCR, T cell receptor; WAS, Wiskott–Aldrich syndrome; HA, hemophilia A; HB, hemophilia B; DBA, Diamond–Blackfan anemia; MDS, myelodysplastic syndromes; AML, acute myeloid leukemia; CML, chronic myeloid leukemia; PNH, paroxysmal nocturnal hemoglobinuria.

## References

[1] Takahashi K., Tanabe K., Ohnuki M., Narita M., Ichisaka T., Tomoda K., Yamanaka S. (2007). Induction of pluripotent stem cells from adult human fibroblasts by defined factors. Cell.

[2] Yu J., Vodyanik M.A., Smuga-Otto K., Antosiewicz-Bourget J., Frane J.L., Tian S., Nie J., Jonsdottir G.A., Ruotti V., Stewart R. (2007). Induced pluripotent stem cell lines derived from human somatic cells. Science.

[3] Sison S.L., Vermilyea S.C., Emborg M.E., Ebert A.D. (2018). Using patient-derived induced pluripotent stem cells to identify Parkinson’s disease-relevant phenotypes. Curr. Neurol. Neurosci. Rep..

[4] Müller M., Seufferlein T., Illing A., Homann J. (2013). Modelling human channelopathies using induced pluripotent stem cells: A comprehensive review. Stem Cells Int..

[5] Xia G., Terada N., Ashizawa T. (2018). Human iPSC models to study orphan diseases: Muscular dystrophies. Curr. Stem Cell Rep..

[6] Takahashi K., Yamanaka S. (2006). Induction of pluripotent stem cells from mouse embryonic and adult fibroblast cultures by defined factors. Cell.

[7] Noguchi H., Miyagi-Shiohira C., Nakashima Y. (2018). Induced tissue-specific stem cells and epigenetic memory in induced pluripotent stem cells. Int. J. Mol. Sci..

[8] Thomson J.A., Itskovitz-Eldor J., Shapiro S.S., Waknitz M.A., Swiergiel J.J., Marshall V.S., Jones J.M. (1998). Embryonic stem cell lines derived from human blastocysts. Science.

[9] Papp B., Plath K. (2011). Reprogramming to pluripotency: Stepwise resetting of the epigenetic landscape. Cell Res..

[10] Kim K., Zhao R., Doi A., Ng K., Unternaehrer J., Cahan P., Huo H., Loh Y.H., Aryee M.J., Lensch M.W. (2011). Donor cell type can influence the epigenome and differentiation potential of human induced pluripotent stem cells. Nat. Biotechnol..

[11] Ohi Y., Qin H., Hong C., Blouin L., Polo J.M., Guo T., Qi Z., Downey S.L., Manos P.D., Rossi D.J. (2011). Incomplete DNA methylation underlies a transcriptional memory of somatic cells in human iPS cells. Nat. Cell Biol..

[12] Polo J.M., Liu S., Figueroa M.E., Kulalert W., Eminli S., Tan K.Y., Apostolou E., Stadtfeld M., Li Y., Shioda T. (2010). Cell type of origin influences the molecular and functional properties of mouse induced pluripotent stem cells. Nat. Biotechnol..

[13] Kim K., Doi A., Wen B., Ng K., Zhao R., Cahan P., Kim J., Aryee M.J., Ji H., Ehrlich L.I. (2010). Epigenetic memory in induced pluripotent stem cells. Nature.

[14] Wang L., Su Y., Huang C., Yin Y., Chu A., Knupp A., Tang Y. (2019). NANOG and LIN28 dramatically improve human cell reprogramming by modulating LIN41 and canonical WNT activities. Biol. Open.

[15] Zhao Y., Yin X., Qin H., Zhu F., Liu H., Yang W., Zhang Q., Xiang C., Hou P., Song Z. (2008). Two supporting factors greatly improve the efficiency of human iPSC generation. Cell Stem Cell.

[16] Han J., Yuan P., Yang H., Zhang J., Soh B.S., Li P., Lim S.L., Cao S., Tay J., Orlov Y.L. (2010). Tbx3 improves the germ-line competency of induced pluripotent stem cells. Nature.

[17] Marión R.M., Strati K., Li H., Murga M., Blanco R., Ortega S., Fernandez-Capetillo O., Serrano M., Blasco M.A. (2009). A p53-mediated DNA damage response limits reprogramming to ensure iPS cell genomic integrity. Nature.

[18] Li H., Collado M., Villasante A., Strati K., Ortega S., Cañamero M., Blasco M.A., Serrano M. (2009). The Ink4/Arf locus is a barrier for iPS cell reprogramming. Nature.

[19] Kawamura T., Suzuki J., Wang Y.V., Menendez S., Morera L.B., Raya A., Wahl G.M., Belmonte J.C.I. (2009). Linking the p53 tumour suppressor pathway to somatic cell reprogramming. Nature.

[20] Huangfu D., Osafune K., Maehr R., Guo W., Eijkelenboom A., Chen S., Muhlestein W., Melton D.A. (2008). Induction of pluripotent stem cells from primary human fibroblasts with only Oct4 and Sox2. Nat. Biotechnol..

[21] Esteban M.A., Wang T., Qin B., Yang J., Qin D., Cai J., Li W., Weng Z., Chen J., Ni S. (2010). Vitamin C enhances the generation of mouse and human induced pluripotent stem cells. Cell Stem Cell.

[22] Kim J.B., Greber B., Araúzo-Bravo M.J., Meyer J., Park K.I., Zaehres H., Schöler H.R. (2009). Direct reprogramming of human neural stem cells by OCT4. Nature.

[23] Robinton D.A., Daley G.Q. (2012). The promise of induced pluripotent stem cells in research and therapy. Nature.

[24] Karagiannis P., Takahashi K., Saito M., Yoshida Y., Okita K., Watanabe A., Inoue H., Yamashita J.K., Todani M., Nakagawa M. (2019). Induced pluripotent stem cells and their use in human models of disease and development. Physiol. Rev..

[25] Wernig M., Lengner C.J., Hanna J., Lodato M.A., Steine E., Foreman R., Staerk J., Markoulaki S., Jaenisch R. (2008). A drug-inducible transgenic system for direct reprogramming of multiple somatic cell types. Nat. Biotechnol..

[26] Schlaeger T.M., Daheron L., Brickler T.R., Entwisle S., Chan K., Cianci A., DeVine A., Ettenger A., Fitzgerald K., Godfrey M. (2015). A comparison of non-integrating reprogramming methods. Nat. Biotechnol..

[27] Fusaki N., Ban H., Nishiyama A., Saeki K., Hasegawa M. (2009). Efficient induction of transgene-free human pluripotent stem cells using a vector based on Sendai virus, an RNA virus that does not integrate into the host genome. Proc. Jpn. Acad. Ser. B Phys. Biol. Sci..

[28] Nishimura K., Sano M., Ohtaka M., Furuta B., Umemura Y., Nakajima Y., Ikehara Y., Kobayashi T., Segawa H., Takayasu S. (2011). Development of defective and persistent Sendai virus vector: A unique gene delivery/expression system ideal for cell reprogramming. J. Biol. Chem..

[29] Ban H., Nishishita N., Fusaki N., Tabata T., Saeki K., Shikamura M., Takada N., Inoue M., Hasegawa M., Kawamata S. (2011). Efficient generation of transgene-free human induced pluripotent stem cells (iPSCs) by temperature-sensitive Sendai virus vectors. Proc. Natl. Acad. Sci. USA.

[30] Ye L., Muench M.O., Fusaki N., Beyer A.I., Wang J., Qi Z., Yu J., Kan Y.W. (2013). Blood cell-derived induced pluripotent stem cells free of reprogramming factors generated by Sendai viral vectors. Stem Cells Transl. Med..

[31] Seki T., Yuasa S., Oda M., Egashira T., Yae K., Kusumoto D., Nakata H., Tohyama S., Hashimoto H., Kodaira M. (2010). Generation of induced pluripotent stem cells from human terminally differentiated circulating T cells. Cell Stem Cell.

[32] Skorik C., Mullin N.K., Shi M., Zhang Y., Hunter P., Tang Y., Hilton B., Schlaeger T.M. (2020). Xeno-free reprogramming of peripheral blood mononuclear erythroblasts on laminin-521. Curr. Protoc. Stem Cell Biol..

[33] Warren L., Manos P.D., Ahfeldt T., Loh Y.H., Li H., Lau F., Ebina W., Mandal P.K., Smith Z.D., Meissner A. (2010). Highly efficient reprogramming to pluripotency and directed differentiation of human cells with synthetic modified mRNA. Cell Stem Cell.

[34] Durruthy-Durruthy J., Briggs S.F., Awe J., Ramathal C.Y., Karumbayaram S., Lee P.C., Heidmann J.D., Clark A., Karakikes I., Loh K.M. (2014). Rapid and efficient conversion of integration-free human induced pluripotent stem cells to GMP-grade culture conditions. PLoS ONE.

[35] Warren L., Lin C. (2019). mRNA-based genetic reprogramming. Mol. Ther..

[36] Kuo C.-H., Ying S.-Y. (2012). Advances in microRNA-mediated reprogramming technology. Stem Cells Int..

[37] Liao B., Bao X., Liu L., Feng S., Zovoilis A., Liu W., Xue Y., Cai J., Guo X., Qin B. (2011). MicroRNA cluster 302-367 enhances somatic cell reprogramming by accelerating a mesenchymal-to-epithelial transition. J. Biol. Chem..

[38] Anokye-Danso F., Trivedi C.M., Juhr D., Gupta M., Cui Z., Tian Y., Zhang Y., Yang W., Gruber P.J., Epstein J.A. (2011). Highly efficient miRNA-mediated reprogramming of mouse and human somatic cells to pluripotency. Cell Stem Cell.

[39] Eminli S., Foudi A., Stadtfeld M., Maherali N., Ahfeldt T., Mostoslavsky G., Hock H., Hochedlinger K. (2009). Differentiation stage determines potential of hematopoietic cells for reprogramming into induced pluripotent stem cells. Nat. Genet..

[40] Chen G., Gulbranson D.R., Hou Z., Bolin J.M., Ruotti V., Probasco M.D., Smuga-Otto K., Howden S.E., Diol N.R., Propson N.E. (2011). Chemically defined conditions for human iPSC derivation and culture. Nat. Methods.

[41] Ludwig T.E., Levenstein M.E., Jones J.M., Berggren W.T., Mitchen E.R., Frane J.L., Crandall L.J., Daigh C.A., Conard K.R., Piekarczyk M.S. (2006). Derivation of human embryonic stem cells in defined conditions. Nat. Biotechnol..

[42] Miyazaki T., Futaki S., Suemori H., Taniguchi Y., Yamada M., Kawasaki M., Hayashi M., Kumagai H., Nakatsuji N., Sekiguchi K. (2012). Laminin E8 fragments support efficient adhesion and expansion of dissociated human pluripotent stem cells. Nat. Commun..

[43] Palis J., Robertson S., Kennedy M., Wall C., Keller G. (1999). Development of erythroid and myeloid progenitors in the yolk sac and embryo proper of the mouse. Development.

[44] Baron M.H., Isern J., Fraser S.T. (2012). The embryonic origins of erythropoiesis in mammals. Blood.

[45] Tober J., Koniski A., McGrath K.E., Vemishetti R., Emerson R., de Mesy-Bentley K.K., Waugh R., Palis J. (2007). The megakaryocyte lineage originates from hemangioblast precursors and is an integral component both of primitive and of definitive hematopoiesis. Blood.

[46] Chen M.J., Li Y., De Obaldia M.E., Yang Q., Yzaguirre A.D., Yamada-Inagawa T., Vink C.S., Bhandoola A., Dzierzak E., Speck N.A. (2011). Erythroid/myeloid progenitors and hematopoietic stem cells originate from distinct populations of endothelial cells. Cell Stem Cell.

[47] Yoshimoto M., Porayette P., Glosson N.L., Conway S.J., Carlesso N., Cardoso A.A., Kaplan M.H., Yoder M.C. (2012). Autonomous murine T-cell progenitor production in the extra-embryonic yolk sac before HSC emergence. Blood.

[48] Lin Y., Yoder M.C., Yoshimoto M. (2014). Lymphoid progenitor emergence in the murine embryo and yolk sac precedes stem cell detection. Stem Cells Dev..

[49] McGrath K.E., Frame J.M., Fegan K.H., Bowen J.R., Conway S.J., Catherman S.C., Kingsley P.D., Koniski A.D., Palis J. (2015). Distinct sources of hematopoietic progenitors emerge before HSCs and provide functional blood cells in the mammalian embryo. Cell Rep..

[50] Medvinsky A., Dzierzak E. (1996). Definitive hematopoiesis is autonomously initiated by the AGM region. Cell.

[51] de Bruijn M.F., Speck N.A., Peeters M.C., Dzierzak E. (2000). Definitive hematopoietic stem cells first develop within the major arterial regions of the mouse embryo. Embo J..

[52] Ivanovs A., Rybtsov S., Welch L., Anderson R.A., Turner M.L., Medvinsky A. (2011). Highly potent human hematopoietic stem cells first emerge in the intraembryonic aorta-gonad-mesonephros region. J. Exp. Med..

[53] Christensen J.L., Wright D.E., Wagers A.J., Weissman I.L. (2004). Circulation and chemotaxis of fetal hematopoietic stem cells. PLoS Biol..

[54] Chadwick K., Wang L., Li L., Menendez P., Murdoch B., Rouleau A., Bhatia M. (2003). Cytokines and BMP-4 promote hematopoietic differentiation of human embryonic stem cells. Blood.

[55] Kennedy M., D’Souza S.L., Lynch-Kattman M., Schwantz S., Keller G. (2007). Development of the hemangioblast defines the onset of hematopoiesis in human ES cell differentiation cultures. Blood.

[56] Pearson S., Sroczynska P., Lacaud G., Kouskoff V. (2008). The stepwise specification of embryonic stem cells to hematopoietic fate is driven by sequential exposure to Bmp4, activin A, bFGF and VEGF. Development.

[57] Pick M., Azzola L., Mossman A., Stanley E.G., Elefanty A.G. (2007). Differentiation of human embryonic stem cells in serum-free medium reveals distinct roles for bone morphogenetic protein 4, vascular endothelial growth factor, stem cell factor, and fibroblast growth factor 2 in hematopoiesis. Stem Cells.

[58] Slukvin I.I. (2013). Hematopoietic specification from human pluripotent stem cells: Current advances and challenges toward de novo generation of hematopoietic stem cells. Blood.

[59] Sturgeon C.M., Ditadi A., Awong G., Kennedy M., Keller G. (2014). Wnt signaling controls the specification of definitive and primitive hematopoiesis from human pluripotent stem cells. Nat. Biotechnol..

[60] Kennedy M., Awong G., Sturgeon C.M., Ditadi A., LaMotte-Mohs R., Zúñiga-Pflücker J.C., Keller G. (2012). T lymphocyte potential marks the emergence of definitive hematopoietic progenitors in human pluripotent stem cell differentiation cultures. Cell Rep..

[61] Sturgeon C.M., Ditadi A., Clarke R.L., Keller G. (2013). Defining the path to hematopoietic stem cells. Nat. Biotechnol..

[62] Demirci S., Tisdale J.F. (2018). Definitive erythropoiesis from pluripotent stem cells: Recent advances and perspectives. Adv. Exp. Med. Biol..

[63] Ran D., Shia W.J., Lo M.C., Fan J.B., Knorr D.A., Ferrell P.I., Ye Z., Yan M., Cheng L., Kaufman D.S. (2013). RUNX1a enhances hematopoietic lineage commitment from human embryonic stem cells and inducible pluripotent stem cells. Blood.

[64] Guo R., Hu F., Weng Q., Lv C., Wu H., Liu L., Li Z., Zeng Y., Bai Z., Zhang M. (2020). Guiding T lymphopoiesis from pluripotent stem cells by defined transcription factors. Cell Res..

[65] Sugimura R., Jha D.K., Han A., Soria-Valles C., da Rocha E.L., Lu Y.F., Goettel J.A., Serrao E., Rowe R.G., Malleshaiah M. (2017). Haematopoietic stem and progenitor cells from human pluripotent stem cells. Nature.

[66] Tan Y.T., Ye L., Xie F., Beyer A.I., Muench M.O., Wang J., Chen Z., Liu H., Chen S.J., Kan Y.W. (2018). Respecifying human iPSC-derived blood cells into highly engraftable hematopoietic stem and progenitor cells with a single factor. Proc. Natl. Acad. Sci. USA.

[67] Amabile G., Welner R.S., Nombela-Arrieta C., D’Alise A.M., Di Ruscio A., Ebralidze A.K., Kraytsberg Y., Ye M., Kocher O., Neuberg D.S. (2013). In vivo generation of transplantable human hematopoietic cells from induced pluripotent stem cells. Blood.

[68] Suzuki N., Yamazaki S., Yamaguchi T., Okabe M., Masaki H., Takaki S., Otsu M., Nakauchi H. (2013). Generation of engraftable hematopoietic stem cells from induced pluripotent stem cells by way of teratoma formation. Mol. Ther..

[69] Tsukada M., Ota Y., Wilkinson A.C., Becker H.J., Osato M., Nakauchi H., Yamazaki S. (2017). In vivo generation of engraftable murine hematopoietic stem cells by Gfi1b, c-Fos, and Gata2 overexpression within teratoma. Stem Cell Rep..

[70] Olivier E.N., Marenah L., McCahill A., Condie A., Cowan S., Mountford J.C. (2016). High-efficiency serum-free feeder-free erythroid differentiation of human pluripotent stem cells using small molecules. Stem Cells Transl. Med..

[71] Fujita A., Uchida N., Haro-Mora J.J., Winkler T., Tisdale J. (2016). β-globin-expressing definitive erythroid progenitor cells generated from embryonic and induced pluripotent stem cell-derived sacs. Stem Cells.

[72] Ochi K., Takayama N., Hirose S., Nakahata T., Nakauchi H., Eto K. (2014). Multicolor staining of globin subtypes reveals impaired globin switching during erythropoiesis in human pluripotent stem cells. Stem Cells Transl. Med..

[73] Yang C.T., Ma R., Axton R.A., Jackson M., Taylor A.H., Fidanza A., Marenah L., Frayne J., Mountford J.C., Forrester L.M. (2017). Activation of KLF1 enhances the differentiation and maturation of red blood cells from human pluripotent stem cells. Stem Cells.

[74] Trakarnsanga K., Wilson M.C., Lau W., Singleton B.K., Parsons S.F., Sakuntanaga P., Kurita R., Nakamura Y., Anstee D.J., Frayne J. (2014). Induction of adult levels of β-globin in human erythroid cells that intrinsically express embryonic or fetal globin by transduction with KLF1 and BCL11A-XL. Haematologica.

[75] Merryweather-Clarke A.T., Tipping A.J., Lamikanra A.A., Fa R., Abu-Jamous B., Tsang H.P., Carpenter L., Robson K.J., Nandi A.K., Roberts D.J. (2016). Distinct gene expression program dynamics during erythropoiesis from human induced pluripotent stem cells compared with adult and cord blood progenitors. BMC Genom..

[76] Dorn I., Klich K., Arauzo-Bravo M.J., Radstaak M., Santourlidis S., Ghanjati F., Radke T.F., Psathaki O.E., Hargus G., Kramer J. (2015). Erythroid differentiation of human induced pluripotent stem cells is independent of donor cell type of origin. Haematologica.

[77] Lapillonne H., Kobari L., Mazurier C., Tropel P., Giarratana M.C., Zanella-Cleon I., Kiger L., Wattenhofer-Donzé M., Puccio H., Hebert N. (2010). Red blood cell generation from human induced pluripotent stem cells: Perspectives for transfusion medicine. Haematologica.

[78] Hansen M., Varga E., Aarts C., Wust T., Kuijpers T., von Lindern M., van den Akker E. (2018). Efficient production of erythroid, megakaryocytic and myeloid cells, using single cell-derived iPSC colony differentiation. Stem Cell Res..

[79] Hansen M., von Lindern M., van den Akker E., Varga E. (2019). Human-induced pluripotent stem cell-derived blood products: State of the art and future directions. FEBS Lett..

[80] Aihara A., Koike T., Abe N., Nakamura S., Sawaguchi A., Nakamura T., Sugimoto N., Nakauchi H., Nishino T., Eto K. (2017). Novel TPO receptor agonist TA-316 contributes to platelet biogenesis from human iPS cells. Blood Adv..

[81] Feng Q., Shabrani N., Thon J.N., Huo H., Thiel A., Machlus K.R., Kim K., Brooks J., Li F., Luo C. (2014). Scalable generation of universal platelets from human induced pluripotent stem cells. Stem Cell Rep..

[82] Moreau T., Evans A.L., Vasquez L., Tijssen M.R., Yan Y., Trotter M.W., Howard D., Colzani M., Arumugam M., Wu W.H. (2016). Large-scale production of megakaryocytes from human pluripotent stem cells by chemically defined forward programming. Nat. Commun..

[83] Ito Y., Nakamura S., Sugimoto N., Shigemori T., Kato Y., Ohno M., Sakuma S., Ito K., Kumon H., Hirose H. (2018). Turbulence activates platelet biogenesis to enable clinical scale ex vivo production. Cell.

[84] Shepherd J.H., Howard D., Waller A.K., Foster H.R., Mueller A., Moreau T., Evans A.L., Arumugam M., Bouët Chalon G., Vriend E. (2018). Structurally graduated collagen scaffolds applied to the ex vivo generation of platelets from human pluripotent stem cell-derived megakaryocytes: Enhancing production and purity. Biomaterials.

[85] Akabayashi A., Nakazawa E., Jecker N.S. (2019). The world’s first clinical trial for an aplastic anemia patient with thrombocytopenia administering platelets generated from autologous iPS cells. Int J. Hematol..

[86] Takata K., Kozaki T., Lee C.Z.W., Thion M.S., Otsuka M., Lim S., Utami K.H., Fidan K., Park D.S., Malleret B. (2017). Induced-pluripotent-stem-cell-derived primitive macrophages provide a platform for modeling tissue-resident macrophage differentiation and function. Immunity.

[87] Lee C.Z.W., Kozaki T., Ginhoux F. (2018). Studying tissue macrophages in vitro: Are iPSC-derived cells the answer?. Nat. Rev. Immunol..

[88] Lachmann N., Ackermann M., Frenzel E., Liebhaber S., Brennig S., Happle C., Hoffmann D., Klimenkova O., Lüttge D., Buchegger T. (2015). Large-scale hematopoietic differentiation of human induced pluripotent stem cells provides granulocytes or macrophages for cell replacement therapies. Stem Cell Rep..

[89] Brauer P.M., Pessach I.M., Clarke E., Rowe J.H., Ott de Bruin L., Lee Y.N., Dominguez-Brauer C., Comeau A.M., Awong G., Felgentreff K. (2016). Modeling altered T-cell development with induced pluripotent stem cells from patients with RAG1-dependent immune deficiencies. Blood.

[90] Nishimura T., Nakauchi H. (2019). Generation of antigen-specific T cells from human induced pluripotent stem cells. Methods Mol. Biol..

[91] Shukla S., Langley M.A., Singh J., Edgar J.M., Mohtashami M., Zúñiga-Pflücker J.C., Zandstra P.W. (2017). Progenitor T-cell differentiation from hematopoietic stem cells using Delta-like-4 and VCAM-1. Nat. Methods.

[92] Iriguchi S., Yasui Y., Kawai Y., Arima S., Kunitomo M., Sato T., Ueda T., Minagawa A., Mishima Y., Yanagawa N. (2021). A clinically applicable and scalable method to regenerate T-cells from iPSCs for off-the-shelf T-cell immunotherapy. Nat. Commun..

[93] Wattanapanitch M., Damkham N., Potirat P., Trakarnsanga K., Janan M., U-Pratya Y., Kheolamai P., Klincumhom N., Issaragrisil S. (2018). One-step genetic correction of hemoglobin E/beta-thalassemia patient-derived iPSCs by the CRISPR/Cas9 system. Stem Cell Res. Ther..

[94] Cai L., Bai H., Mahairaki V., Gao Y., He C., Wen Y., Jin Y.C., Wang Y., Pan R.L., Qasba A. (2018). A universal approach to correct various HBB gene mutations in human stem cells for gene therapy of beta-thalassemia and sickle cell disease. Stem Cells Transl. Med..

[95] Niu X., He W., Song B., Ou Z., Fan D., Chen Y., Fan Y., Sun X. (2016). Combining single strand oligodeoxynucleotides and CRISPR/Cas9 to correct gene mutations in β-thalassemia-induced pluripotent stem cells. J. Biol. Chem..

[96] Wang Y., Zheng C.G., Jiang Y., Zhang J., Chen J., Yao C., Zhao Q., Liu S., Chen K., Du J. (2012). Genetic correction of β-thalassemia patient-specific iPS cells and its use in improving hemoglobin production in irradiated SCID mice. Cell Res..

[97] Liu Y., Yang Y., Kang X., Lin B., Yu Q., Song B., Gao G., Chen Y., Sun X., Li X. (2017). One-step biallelic and scarless correction of a β-thalassemia mutation in patient-specific iPSCs without drug selection. Mol. Ther. Nucleic Acids.

[98] Xiong Z., Xie Y., Yang Y., Xue Y., Wang D., Lin S., Chen D., Lu D., He L., Song B. (2019). Efficient gene correction of an aberrant splice site in β-thalassaemia iPSCs by CRISPR/Cas9 and single-strand oligodeoxynucleotides. J. Cell. Mol. Med..

[99] Ma N., Liao B., Zhang H., Wang L., Shan Y., Xue Y., Huang K., Chen S., Zhou X., Chen Y. (2013). Transcription activator-like effector nuclease (TALEN)-mediated gene correction in integration-free β-thalassemia induced pluripotent stem cells. J. Biol. Chem..

[100] Ma N., Shan Y., Liao B., Kong G., Wang C., Huang K., Zhang H., Cai X., Chen S., Pei D. (2015). Factor-induced reprogramming and zinc finger nuclease-aided gene targeting cause different genome instability in β-thalassemia induced pluripotent stem cells (iPSCs). J. Biol. Chem..

[101] Xu P., Tong Y., Liu X.Z., Wang T.T., Cheng L., Wang B.Y., Lv X., Huang Y., Liu D.P. (2015). Both TALENs and CRISPR/Cas9 directly target the HBB IVS2-654 (C > T) mutation in β-thalassemia-derived iPSCs. Sci. Rep..

[102] Chang C.J., Bouhassira E.E. (2012). Zinc-finger nuclease-mediated correction of α-thalassemia in iPS cells. Blood.

[103] Garate Z., Quintana-Bustamante O., Crane A.M., Olivier E., Poirot L., Galetto R., Kosinski P., Hill C., Kung C., Agirre X. (2015). Generation of a high number of healthy erythroid cells from gene-edited pyruvate kinase deficiency patient-specific induced pluripotent stem cells. Stem Cell Rep..

[104] Kohara H., Utsugisawa T., Sakamoto C., Hirose L., Ogawa Y., Ogura H., Sugawara A., Liao J., Aoki T., Iwasaki T. (2019). KLF1 mutation E325K induces cell cycle arrest in erythroid cells differentiated from congenital dyserythropoietic anemia patient-specific induced pluripotent stem cells. Exp. Hematol..

[105] Zou J., Mali P., Huang X., Dowey S.N., Cheng L. (2011). Site-specific gene correction of a point mutation in human iPS cells derived from an adult patient with sickle cell disease. Blood.

[106] Ramalingam S., Annaluru N., Kandavelou K., Chandrasegaran S. (2014). TALEN-mediated generation and genetic correction of disease-specific human induced pluripotent stem cells. Curr. Gene Ther..

[107] Sun N., Zhao H. (2014). Seamless correction of the sickle cell disease mutation of the HBB gene in human induced pluripotent stem cells using TALENs. Biotechnol. Bioeng..

[108] Huang X., Wang Y., Yan W., Smith C., Ye Z., Wang J., Gao Y., Mendelsohn L., Cheng L. (2015). Production of gene-corrected adult beta globin protein in human erythrocytes differentiated from patient iPSCs after genome editing of the sickle point mutation. Stem Cells.

[109] Park S., Gianotti-Sommer A., Molina-Estevez F.J., Vanuytsel K., Skvir N., Leung A., Rozelle S.S., Shaikho E.M., Weir I., Jiang Z. (2017). A comprehensive, ethnically diverse library of sickle cell disease-specific induced pluripotent stem cells. Stem Cell Rep..

[110] Zou J., Sweeney C.L., Chou B.K., Choi U., Pan J., Wang H., Dowey S.N., Cheng L., Malech H.L. (2011). Oxidase-deficient neutrophils from X-linked chronic granulomatous disease iPS cells: Functional correction by zinc finger nuclease-mediated safe harbor targeting. Blood.

[111] Jiang Y., Cowley S.A., Siler U., Melguizo D., Tilgner K., Browne C., Dewilton A., Przyborski S., Saretzki G., James W.S. (2012). Derivation and functional analysis of patient-specific induced pluripotent stem cells as an in vitro model of chronic granulomatous disease. Stem Cells.

[112] Merling R.K., Sweeney C.L., Chu J., Bodansky A., Choi U., Priel D.L., Kuhns D.B., Wang H., Vasilevsky S., De Ravin S.S. (2015). An AAVS1-targeted minigene platform for correction of iPSCs from all five types of chronic granulomatous disease. Mol. Ther..

[113] Flynn R., Grundmann A., Renz P., Hänseler W., James W.S., Cowley S.A., Moore M.D. (2015). CRISPR-mediated genotypic and phenotypic correction of a chronic granulomatous disease mutation in human iPS cells. Exp. Hematol..

[114] Dreyer A.K., Hoffmann D., Lachmann N., Ackermann M., Steinemann D., Timm B., Siler U., Reichenbach J., Grez M., Moritz T. (2015). TALEN-mediated functional correction of X-linked chronic granulomatous disease in patient-derived induced pluripotent stem cells. Biomaterials.

[115] Laugsch M., Rostovskaya M., Velychko S., Richter C., Zimmer A., Klink B., Schröck E., Haase M., Neumann K., Thieme S. (2016). Functional restoration of gp91phox-oxidase activity by BAC transgenesis and gene targeting in X-linked chronic granulomatous disease iPSCs. Mol. Ther..

[116] Merling R.K., Kuhns D.B., Sweeney C.L., Wu X., Burkett S., Chu J., Lee J., Koontz S., Di Pasquale G., Afione S.A. (2017). Gene-edited pseudogene resurrection corrects p47(phox)-deficient chronic granulomatous disease. Blood Adv..

[117] Klatt D., Cheng E., Philipp F., Selich A., Dahlke J., Schmidt R.E., Schott J.W., Büning H., Hoffmann D., Thrasher A.J. (2019). Targeted repair of p47-CGD in iPSCs by CRISPR/Cas9: Functional correction without cleavage in the highly homologous pseudogenes. Stem Cell Rep..

[118] Menon T., Firth A.L., Scripture-Adams D.D., Galic Z., Qualls S.J., Gilmore W.B., Ke E., Singer O., Anderson L.S., Bornzin A.R. (2015). Lymphoid regeneration from gene-corrected SCID-X1 subject-derived iPSCs. Cell Stem Cell.

[119] Chang C.W., Lai Y.S., Westin E., Khodadadi-Jamayran A., Pawlik K.M., Lamb L.S., Goldman F.D., Townes T.M. (2015). Modeling human severe combined immunodeficiency and correction by CRISPR/Cas9-enhanced gene targeting. Cell Rep..

[120] Themeli M., Chhatta A., Boersma H., Prins H.J., Cordes M., de Wilt E., Farahani A.S., Vandekerckhove B., van der Burg M., Hoeben R.C. (2020). iPSC-based modeling of RAG2 severe combined immunodeficiency reveals multiple T cell developmental arrests. Stem Cell Rep..

[121] Ingrungruanglert P., Amarinthnukrowh P., Rungsiwiwut R., Maneesri-le Grand S., Sosothikul D., Suphapeetiporn K., Israsena N., Shotelersuk V. (2015). Wiskott-Aldrich syndrome iPS cells produce megakaryocytes with defects in cytoskeletal rearrangement and proplatelet formation. Thromb. Haemost..

[122] Laskowski T.J., Van Caeneghem Y., Pourebrahim R., Ma C., Ni Z., Garate Z., Crane A.M., Li X.S., Liao W., Gonzalez-Garay M. (2016). Gene correction of iPSCs from a Wiskott-Aldrich syndrome patient normalizes the lymphoid developmental and functional defects. Stem Cell Rep..

[123] Park C.Y., Kim J., Kweon J., Son J.S., Lee J.S., Yoo J.E., Cho S.R., Kim J.H., Kim J.S., Kim D.W. (2014). Targeted inversion and reversion of the blood coagulation factor 8 gene in human iPS cells using TALENs. Proc. Natl. Acad. Sci. USA.

[124] Wu Y., Hu Z., Li Z., Pang J., Feng M., Hu X., Wang X., Lin-Peng S., Liu B., Chen F. (2016). In situ genetic correction of F8 intron 22 inversion in hemophilia A patient-specific iPSCs. Sci. Rep..

[125] Park C.Y., Kim D.H., Son J.S., Sung J.J., Lee J., Bae S., Kim J.H., Kim D.W., Kim J.S. (2015). Functional correction of large factor VIII gene chromosomal inversions in hemophilia A patient-derived iPSCs using CRISPR-Cas9. Cell Stem Cell.

[126] Pang J., Wu Y., Li Z., Hu Z., Wang X., Hu X., Wang X., Liu X., Zhou M., Liu B. (2016). Targeting of the human F8 at the multicopy rDNA locus in hemophilia A patient-derived iPSCs using TALENickases. Biochem. Biophys. Res. Commun..

[127] Olgasi C., Talmon M., Merlin S., Cucci A., Richaud-Patin Y., Ranaldo G., Colangelo D., Di Scipio F., Berta G.N., Borsotti C. (2018). Patient-specific iPSC-derived endothelial cells provide long-term phenotypic correction of hemophilia A. Stem Cell Rep..

[128] Ramaswamy S., Tonnu N., Menon T., Lewis B.M., Green K.T., Wampler D., Monahan P.E., Verma I.M. (2018). Autologous and heterologous cell therapy for hemophilia B toward functional restoration of factor IX. Cell Rep..

[129] He Q., Wang H.H., Cheng T., Yuan W.P., Ma Y.P., Jiang Y.P., Ren Z.H. (2017). Genetic correction and hepatic differentiation of hemophilia B-specific human induced pluripotent stem cells. Chin. Med. Sci. J..

[130] Lyu C., Shen J., Wang R., Gu H., Zhang J., Xue F., Liu X., Liu W., Fu R., Zhang L. (2018). Targeted genome engineering in human induced pluripotent stem cells from patients with hemophilia B using the CRISPR-Cas9 system. Stem Cell Res. Ther..

[131] Garçon L., Ge J., Manjunath S.H., Mills J.A., Apicella M., Parikh S., Sullivan L.M., Podsakoff G.M., Gadue P., French D.L. (2013). Ribosomal and hematopoietic defects in induced pluripotent stem cells derived from Diamond Blackfan anemia patients. Blood.

[132] Doulatov S., Vo L.T., Macari E.R., Wahlster L., Kinney M.A., Taylor A.M., Barragan J., Gupta M., McGrath K., Lee H.Y. (2017). Drug discovery for Diamond-Blackfan anemia using reprogrammed hematopoietic progenitors. Sci Transl Med..

[133] Ge J., Apicella M., Mills J.A., Garçon L., French D.L., Weiss M.J., Bessler M., Mason P.J. (2015). Dysregulation of the transforming growth factor β pathway in induced pluripotent stem cells generated from patients with Diamond Blackfan anemia. PLoS ONE.

[134] Kotini A.G., Chang C.J., Boussaad I., Delrow J.J., Dolezal E.K., Nagulapally A.B., Perna F., Fishbein G.A., Klimek V.M., Hawkins R.D. (2015). Functional analysis of a chromosomal deletion associated with myelodysplastic syndromes using isogenic human induced pluripotent stem cells. Nat. Biotechnol..

[135] Kotini A.G., Chang C.J., Chow A., Yuan H., Ho T.C., Wang T., Vora S., Solovyov A., Husser C., Olszewska M. (2017). Stage-specific human induced pluripotent stem cells map the progression of myeloid transformation to transplantable leukemia. Cell Stem Cell.

[136] Chao M.P., Gentles A.J., Chatterjee S., Lan F., Reinisch A., Corces M.R., Xavy S., Shen J., Haag D., Chanda S. (2017). Human AML-iPSCs reacquire leukemic properties after differentiation and model clonal variation of disease. Cell Stem Cell.

[137] Bedel A., Pasquet J.M., Lippert E., Taillepierre M., Lagarde V., Dabernat S., Dubus P., Charaf L., Beliveau F., de Verneuil H. (2013). Variable behavior of iPSCs derived from CML patients for response to TKI and hematopoietic differentiation. PLoS ONE.

[138] Suknuntha K., Ishii Y., Tao L., Hu K., McIntosh B.E., Yang D., Swanson S., Stewart R., Wang J.Y.J., Thomson J. (2015). Discovery of survival factor for primitive chronic myeloid leukemia cells using induced pluripotent stem cells. Stem Cell Res..

[139] Charaf L., Mahon F.X., Lamrissi-Garcia I., Moranvillier I., Beliveau F., Cardinaud B., Dabernat S., de Verneuil H., Moreau-Gaudry F., Bedel A. (2017). Effect of tyrosine kinase inhibitors on stemness in normal and chronic myeloid leukemia cells. Leukemia.

[140] Toofan P., Busch C., Morrison H., O’Brien S., Jørgensen H., Copland M., Wheadon H. (2018). Chronic myeloid leukaemia cells require the bone morphogenic protein pathway for cell cycle progression and self-renewal. Cell Death Dis..

[141] Miyauchi M., Koya J., Arai S., Yamazaki S., Honda A., Kataoka K., Yoshimi A., Taoka K., Kumano K., Kurokawa M. (2018). ADAM8 is an antigen of tyrosine kinase inhibitor-resistant chronic myeloid leukemia cells identified by patient-derived induced pluripotent stem cells. Stem Cell Rep..

[142] Yuan X., Braunstein E.M., Ye Z., Liu C.F., Chen G., Zou J., Cheng L., Brodsky R.A. (2013). Generation of glycosylphosphatidylinositol anchor protein-deficient blood cells from human induced pluripotent stem cells. Stem Cells Transl. Med..

[143] Xie F., Ye L., Chang J.C., Beyer A.I., Wang J., Muench M.O., Kan Y.W. (2014). Seamless gene correction of β-thalassemia mutations in patient-specific iPSCs using CRISPR/Cas9 and piggyBac. Genome Res..

[144] Yang Y., Zhang X., Yi L., Hou Z., Chen J., Kou X., Zhao Y., Wang H., Sun X.F., Jiang C. (2016). Naïve induced pluripotent stem cells generated from β-thalassemia fibroblasts allow efficient gene correction with CRISPR/Cas9. Stem Cells Transl. Med..

[145] Potirat P., Wattanapanitch M., Viprakasit V., Kheolamai P., Issaragrisil S. (2019). An integration-free iPSC line (MUSIi008-A) derived from a patient with severe hemolytic anemia carrying compound heterozygote mutations in KLF1 gene for disease modeling. Stem Cell Res..

[146] Hsu J., Reilly A., Hayes B.J., Clough C.A., Konnick E.Q., Torok-Storb B., Gulsuner S., Wu D., Becker P.S., Keel S.B. (2019). Reprogramming identifies functionally distinct stages of clonal evolution in myelodysplastic syndromes. Blood.

[147] Wang T., Pine A.R., Kotini A.G., Yuan H., Zamparo L., Starczynowski D.T., Leslie C., Papapetrou E.P. (2021). Sequential CRISPR gene editing in human iPSCs charts the clonal evolution of myeloid leukemia and identifies early disease targets. Cell Stem Cell.

[148] Ye Z., Zhan H., Mali P., Dowey S., Williams D.M., Jang Y.Y., Dang C.V., Spivak J.L., Moliterno A.R., Cheng L. (2009). Human-induced pluripotent stem cells from blood cells of healthy donors and patients with acquired blood disorders. Blood.

[149] Nilsri N., Jangprasert P., Pawinwongchai J., Israsena N., Rojnuckarin P. (2021). Distinct effects of V617F and exon12-mutated JAK2 expressions on erythropoiesis in a human induced pluripotent stem cell (iPSC)-based model. Sci. Rep..

[150] Saliba J., Hamidi S., Lenglet G., Langlois T., Yin J., Cabagnols X., Secardin L., Legrand C., Galy A., Opolon P. (2013). Heterozygous and homozygous JAK2(V617F) states modeled by induced pluripotent stem cells from myeloproliferative neoplasm patients. PLoS ONE.

[151] Takei H., Edahiro Y., Mano S., Masubuchi N., Mizukami Y., Imai M., Morishita S., Misawa K., Ochiai T., Tsuneda S. (2018). Skewed megakaryopoiesis in human induced pluripotent stem cell-derived haematopoietic progenitor cells harbouring calreticulin mutations. Br. J. Haematol..

[152] Secardin L., Gomez Limia C., da Silva-Benedito S., Lordier L., El-Khoury M., Marty C., Ianotto J.C., Raslova H., Constantinescu S.N., Bonamino M.H. (2021). Induced pluripotent stem cells enable disease modeling and drug screening in calreticulin del52 and ins5 myeloproliferative Neoplasms. Hemasphere.

[153] Brault J., Vaganay G., Le Roy A., Lenormand J.L., Cortes S., Stasia M.J. (2017). Therapeutic effects of proteoliposomes on X-linked chronic granulomatous disease: Proof of concept using macrophages differentiated from patient-specific induced pluripotent stem cells. Int. J. Nanomed..

[154] Liu G.H., Suzuki K., Li M., Qu J., Montserrat N., Tarantino C., Gu Y., Yi F., Xu X., Zhang W. (2014). Modelling Fanconi anemia pathogenesis and therapeutics using integration-free patient-derived iPSCs. Nat. Commun..

[155] Ruiz-Gutierrez M., Bölükbaşı Ö.V., Alexe G., Kotini A.G., Ballotti K., Joyce C.E., Russell D.W., Stegmaier K., Myers K., Novina C.D. (2019). Therapeutic discovery for marrow failure with MDS predisposition using pluripotent stem cells. JCI Insight.

[156] Qanash H., Li Y., Smith R.H., Linask K., Young-Baird S., Hakami W., Keyvanfar K., Choy J.S., Zou J., Larochelle A. (2021). Eltrombopag improves erythroid differentiation in a human induced pluripotent stem cell model of Diamond Blackfan anemia. Cells.

[157] Taoka K., Arai S., Kataoka K., Hosoi M., Miyauchi M., Yamazaki S., Honda A., Aixinjueluo W., Kobayashi T., Kumano K. (2018). Using patient-derived iPSCs to develop humanized mouse models for chronic myelomonocytic leukemia and therapeutic drug identification, including liposomal clodronate. Sci. Rep..

[158] Chang C.J., Kotini A.G., Olszewska M., Georgomanoli M., Teruya-Feldstein J., Sperber H., Sanchez R., DeVita R., Martins T.J., Abdel-Wahab O. (2018). Dissecting the contributions of cooperating gene mutations to cancer phenotypes and drug responses with patient-derived iPSCs. Stem Cell Rep..

[159] Sullivan S., Stacey G.N., Akazawa C., Aoyama N., Baptista R., Bedford P., Bennaceur Griscelli A., Chandra A., Elwood N., Girard M. (2018). Quality control guidelines for clinical-grade human induced pluripotent stem cell lines. Regen. Med..

[160] Barker J.N., Kurtzberg J., Ballen K., Boo M., Brunstein C., Cutler C., Horwitz M., Milano F., Olson A., Spellman S. (2017). Optimal practices in unrelated donor cord blood transplantation for hematologic malignancies. Biol. Blood Marrow Transpl..

[161] Phondeechareon T., Wattanapanitch M., U-Pratya Y., Damkham C., Klincumhom N., Lorthongpanich C., Kheolamai P., Laowtammathron C., Issaragrisil S. (2016). Generation of induced pluripotent stem cells as a potential source of hematopoietic stem cells for transplant in PNH patients. Ann. Hematol..

[162] Mandai M., Watanabe A., Kurimoto Y., Hirami Y., Morinaga C., Daimon T., Fujihara M., Akimaru H., Sakai N., Shibata Y. (2017). Autologous induced stem-cell-derived retinal cells for macular degeneration. N. Engl. J. Med..

[163] Nakamura S., Takayama N., Hirata S., Seo H., Endo H., Ochi K., Fujita K., Koike T., Harimoto K., Dohda T. (2014). Expandable megakaryocyte cell lines enable clinically applicable generation of platelets from human induced pluripotent stem cells. Cell Stem Cell.

[164] Umekage M., Sato Y., Takasu N. (2019). Overview: An iPS cell stock at CiRA. Inflamm. Regen..

[165] Turner M., Leslie S., Martin N.G., Peschanski M., Rao M., Taylor C.J., Trounson A., Turner D., Yamanaka S., Wilmut I. (2013). Toward the development of a global induced pluripotent stem cell library. Cell Stem Cell.

[166] Xu H., Wang B., Ono M., Kagita A., Fujii K., Sasakawa N., Ueda T., Gee P., Nishikawa M., Nomura M. (2019). Targeted disruption of HLA genes via CRISPR-Cas9 generates iPSCs with enhanced immune compatibility. Cell Stem Cell.

[167] Börger A.K., Eicke D., Wolf C., Gras C., Aufderbeck S., Schulze K., Engels L., Eiz-Vesper B., Schambach A., Guzman C.A. (2016). Generation of HLA-universal iPSC-derived megakaryocytes and platelets for survival under refractoriness conditions. Mol. Med..

[168] Mattapally S., Pawlik K.M., Fast V.G., Zumaquero E., Lund F.E., Randall T.D., Townes T.M., Zhang J. (2018). Human leukocyte antigen class I and II knockout human induced pluripotent stem cell-derived cells: Universal donor for cell therapy. J. Am. Heart Assoc..

[169] Deuse T., Hu X., Gravina A., Wang D., Tediashvili G., De C., Thayer W.O., Wahl A., Garcia J.V., Reichenspurner H. (2019). Hypoimmunogenic derivatives of induced pluripotent stem cells evade immune rejection in fully immunocompetent allogeneic recipients. Nat. Biotechnol..

[170] Suzuki D., Flahou C., Yoshikawa N., Stirblyte I., Hayashi Y., Sawaguchi A., Akasaka M., Nakamura S., Higashi N., Xu H. (2020). iPSC-derived platelets depleted of HLA class I are inert to anti-HLA class I and natural killer cell immunity. Stem Cell Rep..

[171] Norbnop P., Ingrungruanglert P., Israsena N., Suphapeetiporn K., Shotelersuk V. (2020). Generation and characterization of HLA-universal platelets derived from induced pluripotent stem cells. Sci. Rep..

[172] Hanna J., Wernig M., Markoulaki S., Sun C.W., Meissner A., Cassady J.P., Beard C., Brambrink T., Wu L.C., Townes T.M. (2007). Treatment of sickle cell anemia mouse model with iPS cells generated from autologous skin. Science.

[173] Li L.B., Ma C., Awong G., Kennedy M., Gornalusse G., Keller G., Kaufman D.S., Russell D.W. (2016). Silent IL2RG gene editing in human pluripotent stem cells. Mol. Ther..

[174] Mullard A. (2021). FDA approves fourth CAR-T cell therapy. Nat. Rev. Drug Discov..

[175] Luanpitpong S., Poohadsuan J., Klaihmon P., Issaragrisil S. (2021). Selective cytotoxicity of single and dual anti-CD19 and anti-CD138 chimeric antigen receptor-natural killer cells against hematologic malignancies. J. Immunol. Res..

[176] Graham C., Jozwik A., Pepper A., Benjamin R. (2018). Allogeneic CAR-T cells: More than ease of access?. Cells.

[177] Pfefferle A., Huntington N.D. (2020). You have got a fast CAR: Chimeric antigen receptor NK cells in cancer therapy. Cancers.

[178] Themeli M., Kloss C.C., Ciriello G., Fedorov V.D., Perna F., Gonen M., Sadelain M. (2013). Generation of tumor-targeted human T lymphocytes from induced pluripotent stem cells for cancer therapy. Nat. Biotechnol..

[179] Zhang J., Zheng H., Diao Y. (2019). Natural killer cells and current applications of chimeric antigen receptor-modified NK-92 cells in tumor immunotherapy. Int. J. Mol. Sci..

[180] Arias J., Yu J., Varshney M., Inzunza J., Nalvarte I. (2021). Hematopoietic stem cell- and induced pluripotent stem cell-derived CAR-NK cells as reliable cell-based therapy solutions. Stem Cells Transl. Med..

[181] Mehta R.S., Rezvani K. (2018). Chimeric antigen receptor expressing natural killer cells for the immunotherapy of cancer. Front. Immunol..

[182] Mirzaei H.R., Rodriguez A., Shepphird J., Brown C.E., Badie B. (2017). Chimeric antigen receptors T cell therapy in solid tumor: Challenges and clinical applications. Front. Immunol..

[183] Martinez M., Moon E.K. (2019). CAR T cells for solid tumors: New strategies for finding, infiltrating, and surviving in the tumor microenvironment. Front. Immunol..

[184] Chen Y., Yu Z., Tan X., Jiang H., Xu Z., Fang Y., Han D., Hong W., Wei W., Tu J. (2021). CAR-macrophage: A new immunotherapy candidate against solid tumors. Biomed. Pharmacother..

[185] Annunziata C.M., Ghobadi A., Pennella E.J., Vanas J., Powell C., Pavelova M., Wagner C., Kuo M., Ullmann C.D., Hassan R. (2020). Feasibility and preliminary safety and efficacy of first-in-human intraperitoneal delivery of MCY-M11, anti-human-mesothelin CAR mRNA transfected into peripheral blood mononuclear cells, for ovarian cancer and malignant peritoneal mesothelioma. J. Clin. Oncol..

[186] Zhang L., Tian L., Dai X., Yu H., Wang J., Lei A., Zhu M., Xu J., Zhao W., Zhu Y. (2020). Pluripotent stem cell-derived CAR-macrophage cells with antigen-dependent anti-cancer cell functions. J. Hematol. Oncol..

[187] Kilpinen H., Goncalves A., Leha A., Afzal V., Alasoo K., Ashford S., Bala S., Bensaddek D., Casale F.P., Culley O.J. (2017). Common genetic variation drives molecular heterogeneity in human iPSCs. Nature.

[188] DeBoever C., Li H., Jakubosky D., Benaglio P., Reyna J., Olson K.M., Huang H., Biggs W., Sandoval E., D’Antonio M. (2017). Large-scale profiling reveals the influence of genetic variation on gene expression in human induced pluripotent stem cells. Cell Stem Cell.

[189] Nishizawa M., Chonabayashi K., Nomura M., Tanaka A., Nakamura M., Inagaki A., Nishikawa M., Takei I., Oishi A., Tanabe K. (2016). Epigenetic variation between human induced pluripotent stem cell lines is an indicator of differentiation capacity. Cell Stem Cell.

[190] Kyttälä A., Moraghebi R., Valensisi C., Kettunen J., Andrus C., Pasumarthy K.K., Nakanishi M., Nishimura K., Ohtaka M., Weltner J. (2016). Genetic variability overrides the impact of parental cell type and determines iPSC differentiation potential. Stem Cell Rep..

[191] Kajiwara M., Aoi T., Okita K., Takahashi R., Inoue H., Takayama N., Endo H., Eto K., Toguchida J., Uemoto S. (2012). Donor-dependent variations in hepatic differentiation from human-induced pluripotent stem cells. Proc. Natl. Acad. Sci. USA.

[192] Theunissen T.W., Powell B.E., Wang H., Mitalipova M., Faddah D.A., Reddy J., Fan Z.P., Maetzel D., Ganz K., Shi L. (2014). Systematic identification of culture conditions for induction and maintenance of naive human pluripotency. Cell Stem Cell.

[193] Takashima Y., Guo G., Loos R., Nichols J., Ficz G., Krueger F., Oxley D., Santos F., Clarke J., Mansfield W. (2014). Resetting transcription factor control circuitry toward ground-state pluripotency in human. Cell.

[194] Canu G., Athanasiadis E., Grandy R.A., Garcia-Bernardo J., Strzelecka P.M., Vallier L., Ortmann D., Cvejic A. (2020). Analysis of endothelial-to-haematopoietic transition at the single cell level identifies cell cycle regulation as a driver of differentiation. Genome Biol..

[195] Rostovskaya M., Bredenkamp N., Smith A. (2015). Towards consistent generation of pancreatic lineage progenitors from human pluripotent stem cells. Philos. Trans. R Soc. Lond. B Biol. Sci..

[196] Lee A.S., Tang C., Cao F., Xie X., van der Bogt K., Hwang A., Connolly A.J., Robbins R.C., Wu J.C. (2009). Effects of cell number on teratoma formation by human embryonic stem cells. Cell Cycle.

[197] Hentze H., Soong P.L., Wang S.T., Phillips B.W., Putti T.C., Dunn N.R. (2009). Teratoma formation by human embryonic stem cells: Evaluation of essential parameters for future safety studies. Stem Cell Res..

[198] Haridass D., Yuan Q., Becker P.D., Cantz T., Iken M., Rothe M., Narain N., Bock M., Nörder M., Legrand N. (2009). Repopulation efficiencies of adult hepatocytes, fetal liver progenitor cells, and embryonic stem cell-derived hepatic cells in albumin-promoter-enhancer urokinase-type plasminogen activator mice. Am. J. Pathol..

[199] Martin R.M., Fowler J.L., Cromer M.K., Lesch B.J., Ponce E., Uchida N., Nishimura T., Porteus M.H., Loh K.M. (2020). Improving the safety of human pluripotent stem cell therapies using genome-edited orthogonal safeguards. Nat. Commun..

[200] Kojima K., Miyoshi H., Nagoshi N., Kohyama J., Itakura G., Kawabata S., Ozaki M., Iida T., Sugai K., Ito S. (2019). Selective ablation of tumorigenic cells following human induced pluripotent stem cell-derived neural stem/progenitor cell transplantation in spinal cord injury. Stem Cells Transl. Med..

